# Comparison of Endovascular Interventions for the Treatment of Superficial Femoral Artery Disease: A Network Meta-analysis

**DOI:** 10.1016/j.jscai.2024.102432

**Published:** 2025-01-21

**Authors:** Andrew W. Schwartz, Yousuf Shah, Haocheng Huang, Ashwin Nathan, Alexander C. Fanaroff, Jay S. Giri, Sahil A. Parikh, Alexandra J. Lansky, Tayyab Shah

**Affiliations:** a Yale Cardiovascular Research Group, Division of Cardiovascular Medicine, Department of Internal Medicine, Yale School of Medicine, New Haven, Connecticut; b Department of Medicine, University of Rochester School of Medicine and Dentistry, Rochester, New York; c Cardiovascular Medicine Division, The Hospital of the University of Pennsylvania, Philadelphia, Pennsylvania; d Division of Cardiology, Columbia University Irving Medical Center, New York, New York

**Keywords:** endovascular, meta-analysis, revascularization, superficial femoral artery

## Abstract

**Background:**

To understand the relative safety and efficacy of endovascular treatment modalities used for superficial femoral artery (SFA) disease, we performed a network meta-analysis to compare outcomes between percutaneous transluminal angioplasty (PTA), atherectomy (A), bare metal stent (BMS), brachytherapy/radiotherapy, covered stent graft (CSG), cutting balloon angioplasty (CBA), drug-coated balloon (DCB), drug-eluting stent (DES), and intravascular lithotripsy (L).

**Methods:**

We performed a systematic literature search of PubMed from January 2000 to January 2023 to identify randomized trials comparing endovascular interventions for the treatment of SFA disease. The primary end points were technical success and 12-month primary patency.

**Results:**

In total, 57 studies (9089 patients) were included. The mean age of the included patients was 68.4 years, 41.4% had diabetes, 18.3% had critical limb ischemia, and 81.3% had de novo lesions. A mean of 1.2 lesions were treated per patient. Technical success was superior for CSG, BMS, and A+DCB compared with PTA, while A+DCB and CSG were superior to DCB. All interventions except brachytherapy alone had superior primary patency compared with PTA. There were no significant differences in 12-month mortality or major amputation. All interventions except L+DCB, PTA+A, and CBA were superior to PTA regarding target lesion revascularization, while only DCB, DES, and BMS were better than PTA at improving Rutherford classification.

**Conclusions:**

In SFA disease, PTA alone is mostly inferior to other endovascular techniques. This comparison of other endovascular techniques will be valuable for endovascular device selection in the treatment of SFA disease.

## Introduction

Peripheral arterial disease (PAD) remains a significant cause of morbidity and mortality worldwide, with increasing prevalence and associated health care costs.^1^^,^^2^ Chronic limb-threatening ischemia (CLTI), the most severe form of PAD, is associated with a significant risk of amputation and death.^3^ Disease of the superficial femoral artery (SFA) is the leading cause of both intermittent claudication (IC) and CLTI.^4^ Treatment of PAD often includes revascularization of the lower extremities, either by endovascular intervention or surgical bypass. Currently there is conflicting randomized evidence on whether surgical bypass or modern endovascular therapy is superior for various cohorts of CLTI patients.^5^^,^^6^ Regardless, endovascular revascularization of the lower extremities is indicated in many patients with SFA disease, including patients needing revascularization for IC resistant to medical management, those without adequate venous grafts, poor surgical candidates, or those who prefer a less invasive endovascular approach.^7^, ^8^, ^9^

The rapid growth of new endovascular therapies and new randomized controlled trials (RCTs) testing their effects in PAD patients has led to a significant amount of new data over the past decade^10^, ^11^, ^12^, ^13^; however, it remains unclear how each novel therapy should fit into real-world practice. Both the American and European guidelines highlight the lack of high quality and comprehensive evidence in this space and call for further data to be gathered.^14^^,^^15^ Thus, understanding the relative safety and efficacy of each intervention will help guide decision making in treating patients and may inform future guidelines. Thus, we conducted a network meta-analysis of RCTs to compare the safety and efficacy of endovascular treatment modalities for SFA disease.

## Methods

### Search strategy

We performed a network meta-analysis following the Preferred Reporting Items for Systematic Reviews and Meta-Analyses (PRISMA) guidelines.^16^ The protocol was registered in PROSPERO (CRD42022377373). A systematic literature search of PubMed was completed including studies from January 2000 to January 2023 to identify RCTs comparing ≥2 endovascular interventions for the treatment of SFA disease that were published in the English language. The specific search terms used were (“femoral” or “femoropopliteal” or “peripheral artery”) AND (“endovascular” OR “angioplasty” OR “balloon” OR “stent” OR “atherectomy” OR “lithotripsy”). The bibliographies of relevant studies were also examined to identify other potentially relevant studies.

### Inclusion/exclusion criteria

The studies were screened by 2 independent authors (A.S. and T.S.) and were included if they were RCTs that compared ≥2 endovascular interventions for the treatment of SFA disease (IC and/or CLTI) in patients aged >18 years. Studies were excluded if they did not report any of the primary or secondary outcomes of this meta-analysis or if they did not have independent core laboratory adjudication of angiographic and/or ultrasound outcomes. Studies that reported on the same trial were included and searched for relevant data and were counted as one study in total. Any discrepancy was resolved by a third reviewer (Y.S.). Endovascular interventions and their combinations included in the study were percutaneous transluminal angioplasty (PTA), percutaneous transluminal angioplasty + atherectomy (PTA+A); drug-coated balloon (DCB), lithotripsy + drug-coated balloon (L+DCB), atherectomy + drug-coated balloon (A+DCB), drug-coated balloon + bare metal stent (DCB+BMS), drug-eluting stent (DES), bare metal stent (BMS), brachytherapy/radiation therapy (brachy), bare metal stent + brachytherapy/radiation therapy (BMS+brachy), covered stent graft (CSG), and cutting balloon angioplasty (CBA).

### Data extraction

For every selected study, 2 authors (A.S., T.S, and/or Y.S.) read the text and extracted study details, sample size, patient demographics and comorbidities, lesion characteristics, and procedural characteristics. Discrepancies were reviewed by the 2 reviewers to reach consensus. Claude-3-Opus (Anthropic) was used to verify data extraction, and any discrepancies were verified and corrected.

### Outcomes

The primary efficacy outcome was 12-month primary patency, defined as freedom from >50% restenosis (peak systolic velocity ratio <2.5 on duplex ultrasound) of the treated lesion and freedom from target lesion revascularization (TLR). The primary procedural outcome was technical success, defined as <30% residual stenosis on immediate postintervention angiogram.

The secondary 12-month safety outcomes were major amputation, defined as any leg amputation proximal to the ankle, all-cause death, and TLR, defined as revascularization of the treated arterial segment. The secondary efficacy outcomes included primary patency at 6 months, 12-month improvement in Rutherford category by ≥1, mean change of Walking Impairment Questionnaire score, and mean change of EuroQol-5 Dimension score, which is a standardized questionnaire to measure health-related quality of life. One-month TLR, 6-month and 12-month late lumen loss, and 1-month all-cause death were prespecified secondary end points, but they were not reported due to the limited number of studies available to include in the network.

### Statistical analysis

Categorical variables, such as patient demographics, are reported as percentages whereas continuous variables are reported as mean ± SD. A network meta-analysis was conducted through simultaneous analysis of direct comparisons of interventions within RCTs and indirect comparison across trials using common comparators. Heterogeneity between studies was assessed using *I*^2^. Random effect models were used, given the intermediate heterogeneity across trials. When available, PTA was used as a control comparator. The effect estimate for each of the outcomes is presented as an odds ratio (OR) with 95% CI. Prediction intervals were also calculated for each primary outcome. P-scores, which measure the certainty that a treatment is better than other treatments, were calculated based on meta-analytic point estimates and standard errors and used to rank order different interventions.^17^

Meta-regression was used to identify if effect sizes of various interventions varied by proportion of various subgroups included in the trials. Subgroups tested included age, female sex, diabetes, current smoking status, chronic kidney disease, end-stage renal disease, CLTI, total occlusions, severe calcification, and de novo lesions. Only results of DCB vs PTA meta-regressions are shown, given the limited number of studies available for other comparisons to conduct meta-regression. We did not account for multiple testing in the primary analysis because the comparisons were only hypothesis-generating and we did not expect to find significant results for most tested subgroups. As a sensitivity analysis we used the Bonferroni method to account for multiple testing.

### Risk of bias

A risk of bias assessment was completed using the Cochrane Risk of Bias 2 tool.^18^ Each study was evaluated in 5 different categories to determine an overall risk of bias, including randomization process, deviation from the intended intervention, missing outcome data, measurement of the outcome, and selection of reported results. Publication bias was assessed by funnel plots and asymmetry tests (Egger’s test).

## Results

### Study selection

**Figure 1:**
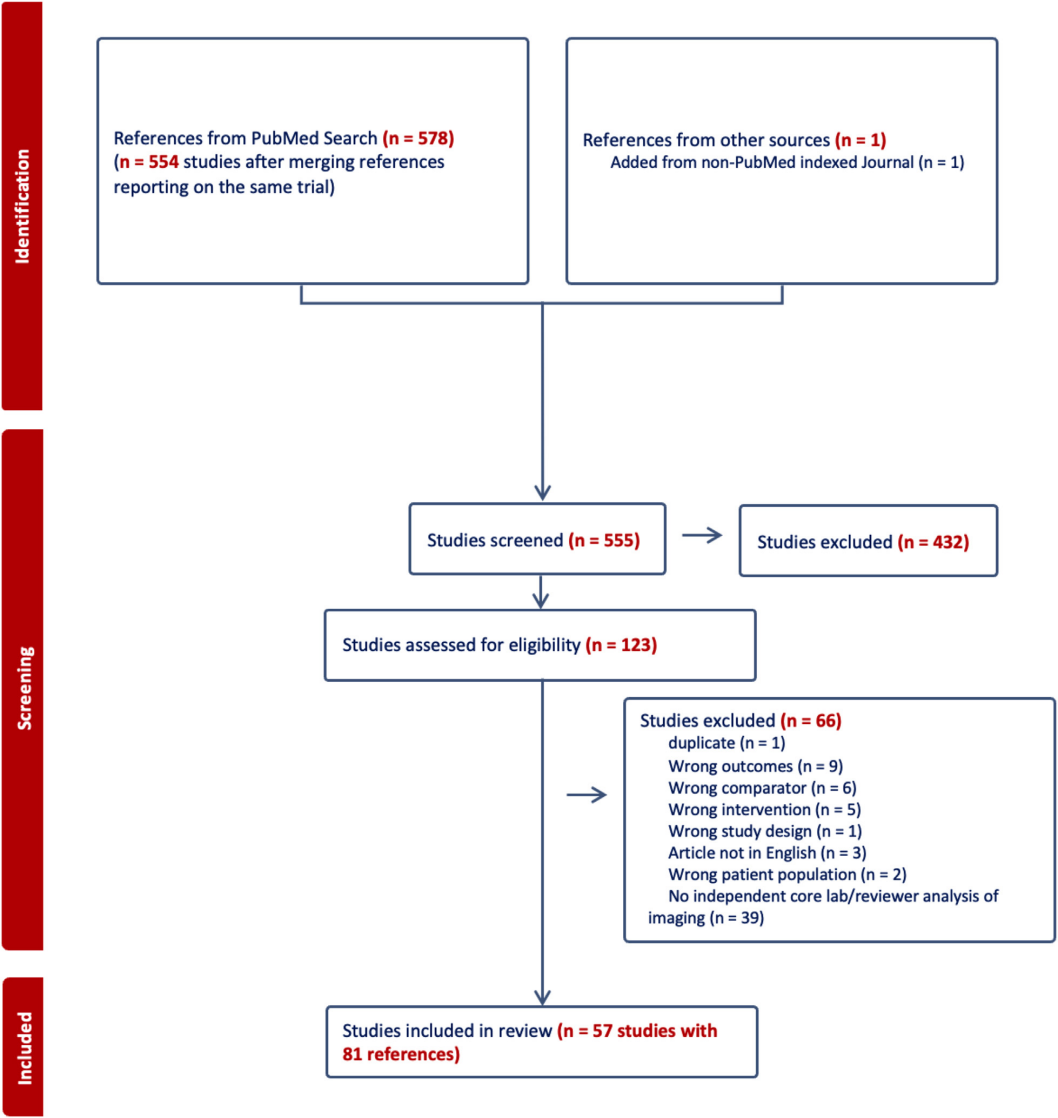
**PRISMA flow diagram of the networkmeta-analysis**.

The search identified 555 total studies with 81 references encompassing 57 studies (9089 total patients) included in the final analysis (Figure 1).^10^^,^^13^^,^^19^, ^20^, ^21^, ^22^, ^23^, ^24^, ^25^, ^26^, ^27^, ^28^, ^29^, ^30^, ^31^, ^32^, ^33^, ^34^, ^35^, ^36^, ^37^, ^38^, ^39^, ^40^, ^41^, ^42^, ^43^, ^44^, ^45^, ^46^, ^47^, ^48^, ^49^, ^50^, ^51^, ^52^, ^53^, ^54^, ^55^, ^56^, ^57^, ^58^, ^59^, ^60^, ^61^, ^62^, ^63^, ^64^, ^65^, ^66^, ^67^, ^68^, ^69^, ^70^, ^71^, ^72^, ^73^ Details of all included studies are presented in Supplemental Table S1. Four of the included studies reported only on secondary outcomes for which there was not enough data to develop a network.^19^^,^^34^^,^^44^^,^^45^ The studies were found to generally be at low risk of bias (Supplemental Table S2) and there was no evidence of publication bias (Supplemental Figures S1 and S2).

### Cohort characteristics

The baseline characteristics of the population are outlined in Table 1. Briefly, the mean age was 68.4 years, 66.7% were male, and 84.4% were White. A total of 41.4% had diabetes mellitus, 41.2% were current smokers, 68.1% had dyslipidemia, 79.3% had hypertension, and 12.8% had renal insufficiency. The mean target leg ankle–brachial index was 0.6, most patients had Rutherford Class 3 (63.1%), and 18.3% had CLTI before intervention. A mean of 1.2 lesions were treated per patient with 81.3% being de novo lesions (Table 2). There was popliteal involvement in 16.6% of lesions treated, and 35.7% of patients had chronic total occlusions. Patient characteristics by each tested endovascular intervention is available in Supplemental Table S3.

**Table 1:** Baseline characteristics.

	N = 7271
Age, y	68.4 ± 9.0
Male sex	66.7%
Body mass index, kg/m^2^	27.3 ± 4.5
Hypertension	79.3%
Dyslipidemia	68.1%
Diabetes mellitus	41.4%
Current smoking	41.2%
Heart failure	5.9%
Coronary artery disease	42.9%
Prior myocardial infarction	18.5%
Carotid artery disease	23.0%
Prior CVA (stroke/TIA)	13.2%
Renal insufficiency	12.8%
End-stage renal disease	6.6%
Target leg ankle–brachial index	0.6 ± 0.2
Rutherford class	
1	4.8%
2	25.0%
3	63.1%
4	9.0%
5	8.3%
6	1.1%
Chronic limb-threatening ischemia (Rutherford 4-6)	18.3%
Prior interventions	46.6%
Percutaneous transluminal angioplasty	16.8%
Drug-coated balloon	1.6%
Stent	39.4%
Atherectomy	2.8%

N = average number of patients with data available from total of 9089. Values are mean ± SD or %.

CVA, cerebrovascular accident; TIA, transient ischemic attack.

**Table 2:** Lesion and procedural characteristics.

Lesion characteristics	N = 7480

No. of lesions treated	1.2 ± 0.9
De novo lesions	81.3%
In-stent restenosis	18.0%
No. of patient runoff vessels	
0	8.6%
1	21.2%
2	37.3%
3	40.1%
Popliteal involvement	16.6%
Lesion length, cm	9.3 ± 5.3
Total occlusion	35.7%
Diameter stenosis at baseline, %	81.7 ± 13.6
Calcification	
None	33.8%
Mild/moderate	46.4%
Severe	21.3%
Procedural characteristics	N = 6526
Procedure time, min	64.7 ± 32.7
No. of treatment balloons	1.3 ± 0.4
Procedural complication	
Any dissection	38.6%
Flow-limiting dissection (≥ type D)	5.2%
Thrombus	3.0%
Aneurysm/pseudoaneurysm	1.9%
Perforation	0.8%
Distal embolus	1.6%
Diameter stenosis after intervention, %	22.4 ± 11.0

N = average number of lesions or patients with relevant data available from total of 9089. Values are mean ± SD or %.

### Primary end points

**Figure 2:**
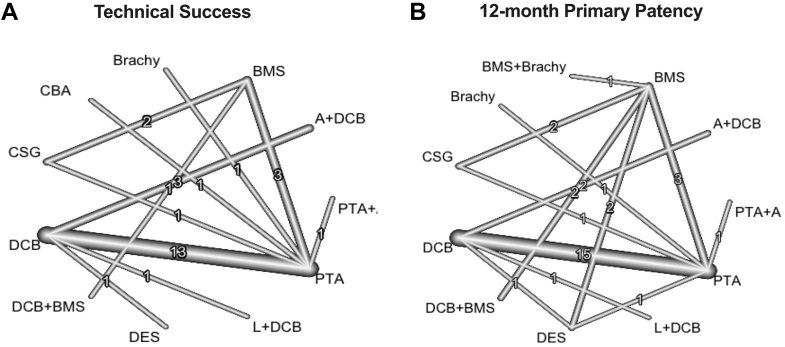
**Network plots for primary outcomes.** Line thickness is weight by amount of randomized controlled trials used to compare the 2 groups by outcome. (**A**) Technical success; (**B**) primary patency.

**Figure 3:**
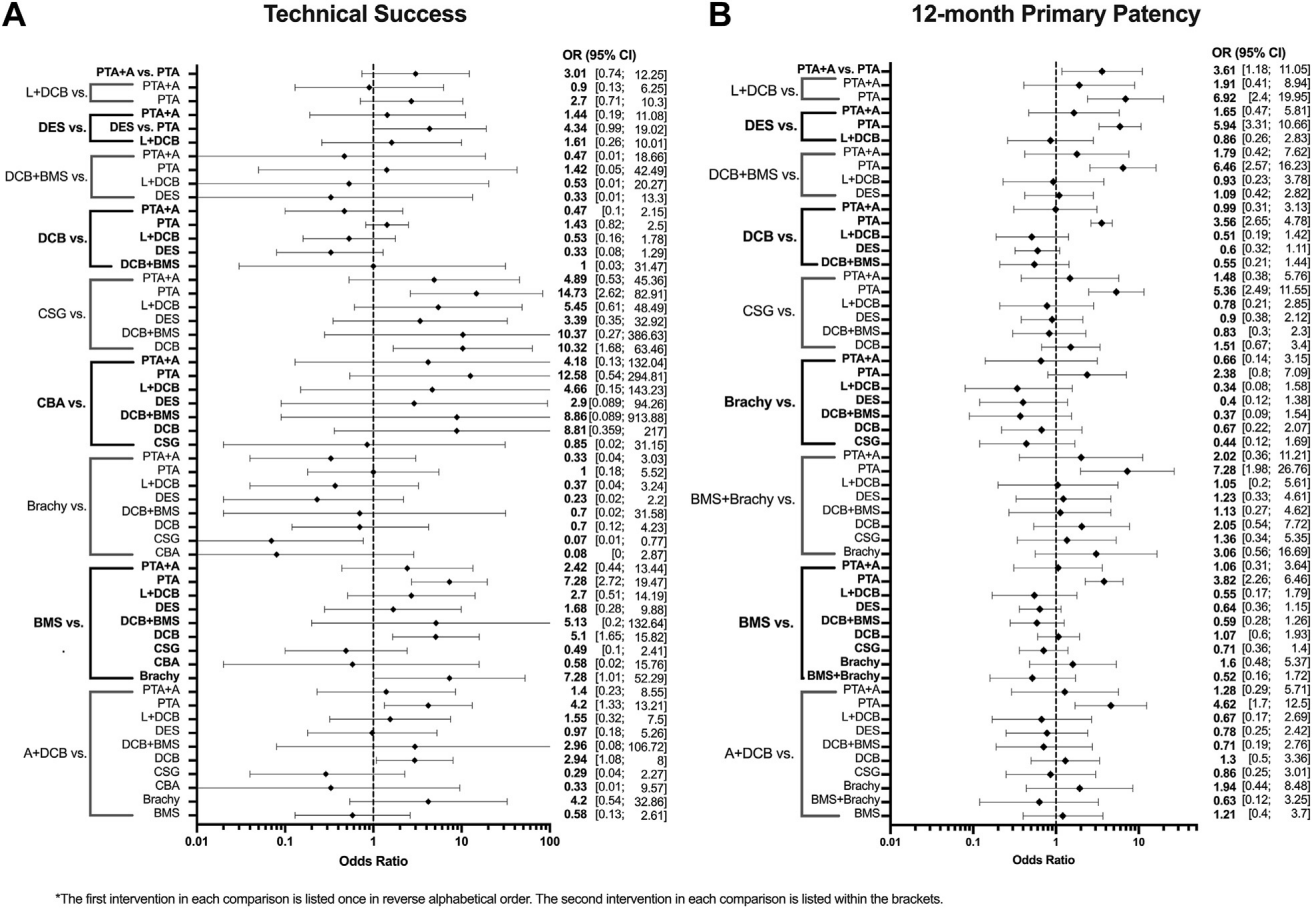
**Primary outcomes of the network meta-analysis.** (**A**) Technical success; (**B**) primary patency at 12 months. Endovascular interventions with their comparators are on the left of the y-axis, with corresponding odds ratio (OR) with 95% CI displayed on the right. A, atherectomy; BMS, bare metal stent; brachy, brachytherapy; CBA, cutting balloon angioplasty; CSG, covered stent graft; DCB, drug-coated balloon; DES, drug-eluting stent; L, lithotripsy; PTA, percutaneous transluminal angioplasty.

Network plots are shown in Figure 2. There were 37 total studies including 5765 patients that assessed technical success among 11 endovascular treatments (Figure 3A).^10^^,^^12^^,^^13^^,^^21^, ^22^, ^23^, ^24^^,^^28^, ^29^, ^30^, ^31^^,^^33^^,^^36^^,^^38^^,^^40^^,^^43^^,^^46^, ^47^, ^48^, ^49^, ^50^, ^51^, ^52^, ^53^^,^^58^, ^59^, ^60^, ^61^, ^62^, ^63^, ^64^^,^^66^^,^^68^, ^69^, ^70^^,^^72^^,^^73^ There was no significant heterogeneity across trials (*I*^2^ = 35.9%; 95% CI, 0.0%-63.1%). Technical success was superior for CSG (OR, 14.73), BMS (OR, 7.28), and A+DCB (OR, 4.2) compared with PTA, whereas A+DCB (OR, 2.94) and CSG (OR, 10.32) were superior to DCB, and BMS (OR, 7.28) was superior to brachy. Technical success ranked highest for CSG (0.88) based on P-scores; the remaining rankings are detailed in Table 3.

**Table 3:** Ranked P-scores for primary and secondary outcomes.

Endovascular intervention	Primary patency 12 mo	Technical success	Target lesion revascularization 12 mo	Rutherford improvement 12 mo	Major amputation 12 mo	All-cause death 12 mo	WIQ score change 12 mo	EQ-5D score change 12 mo
BMS+brachy	0.75	–	–	–	–	–	–	–
L+DCB	0.74	0.47	0.27	–	–	–	–	–
DCB+BMS	0.73	0.34	0.74	0.99	0.2	0.59	–	–
DES	0.71	0.61	0.44	0.78	0.62	0.33	–	–
CSG	0.63	0.88	0.66	0.51	–	0.25	–	–
A+DCB	0.54	0.61	0.89	0.17	–	0.48	–	0.55
PTA+A	0.42	0.50	0.33	–	–	0.46	–	–
BMS	0.39	0.76	0.33	0.66	0.59	0.47	–	–
DCB	0.35	0.25	0.68	0.49	0.53	0.49	0.93	0.65
Brachy	0.24	0.19	0.46	0.2	–	–	–	–
PTA	0.01	0.12	0.05	0.2	0.55	0.46	0.06	0.29
CBA	–	0.74	0.63	–	–	–	–	–

Ranked P-scores for primary and secondary outcomes. P-scores indicate the certainty an intervention is superior than other interventions for the outcome.

A, atherectomy; BMS, bare metal stent; brachy, brachytherapy; CBA, cutting balloon angioplasty; CSG, covered stent graft; DCB, drug-coated balloon; DES, drug-eluting stent; EQ-5D, EuroQol 5-Dimension; L, lithotripsy; PTA, percutaneous transluminal angioplasty; WIQ, Walking Impairment Questionnaire.

A total of 33 studies including 5907 patients that evaluated 11 different endovascular treatments assessed 12-month primary patency (Figure 3B).^10^^,^^13^^,^^20^, ^21^, ^22^, ^23^^,^^25^^,^^26^^,^^28^^,^^30^^,^^31^^,^^33^^,^^36^^,^^38^^,^^43^^,^^47^^,^^48^^,^^50^^,^^51^^,^^56^, ^57^, ^58^, ^59^, ^60^^,^^62^, ^63^, ^64^, ^65^, ^66^^,^^68^^,^^71^, ^72^, ^73^ There was significant heterogeneity across trials (*I*^2^ = 57.6%; 95% CI, 33.2%-73.0%). Primary patency was superior for L+DCB (OR, 6.92), DES (OR, 5.94), DCB+BMS (OR, 6.46), DCB (OR, 3.65), CSG (OR, 5.36), BMS+brachy (OR, 7.28), BMS (OR, 3.82), and A+DCB (OR, 4.62) compared with PTA. BMS+brachy (0.75), L+DCB (0.74), DCB+BMS (0.73), and DES (0.71) ranked highest by P-scores (Table 3). Results were similar for 6-month primary patency (Supplemental Figure S3A). Prediction intervals for primary end points, using PTA as a common comparator, showed similar trends to CIs and are available in Supplemental Table S4.

### Secondary end points

**Figure 4:**
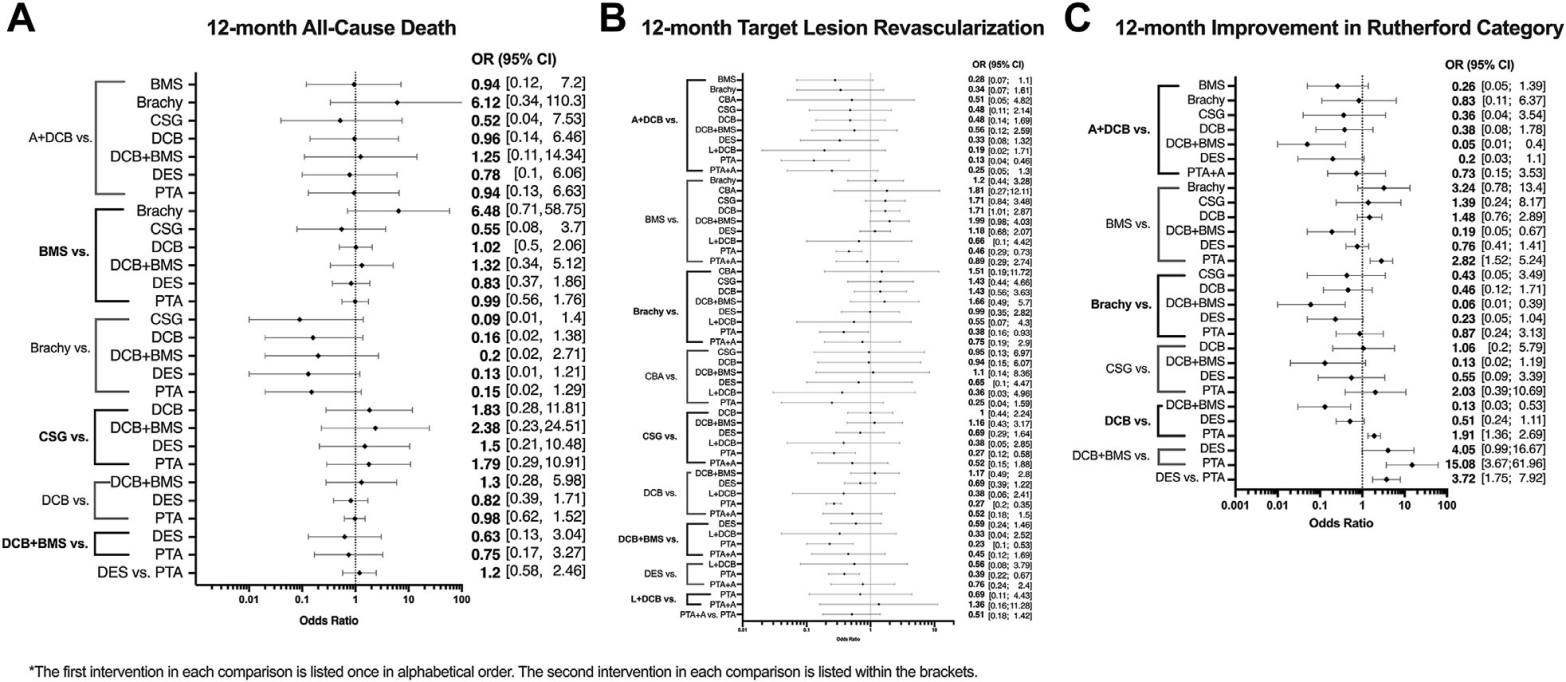
**Secondary outcomes of the network meta-analysis.** (**A**) All-cause death at 12 months; (**B**) target lesion revascularization at 12 months; (**C**) improvement in Rutherford category at 12 months. Endovascular interventions with their comparators are on the left of the y-axis, with corresponding odds ratio (OR) with 95% CI displayed on the right. A, atherectomy; BMS, bare metal stent; Brachy, brachytherapy; CBA, cutting balloon angioplasty; CSG, covered stent graft; DCB, drug-coated balloon; DES, drug-eluting stent; L, lithotripsy; PTA, percutaneous transluminal angioplasty.

Network plots are shown in Supplemental Figure S4. A total of 37 studies including 6556 patients assessed 12-month mortality for 9 endovascular interventions.^12^^,^^23^, ^24^, ^25^, ^26^^,^^28^, ^29^, ^30^, ^31^, ^32^, ^33^^,^^35^, ^36^, ^37^^,^^42^^,^^47^, ^48^, ^49^, ^50^, ^51^, ^52^^,^^56^^,^^57^^,^^59^, ^60^, ^61^, ^62^, ^63^, ^64^, ^65^, ^66^, ^67^, ^68^, ^69^, ^70^, ^71^^,^^73^ Mortality (Figure 4A) and 12-month major amputation (Supplementary Figure S3B) were not different across treatments.

A total of 49 total studies including 7801 patients assessed 12-month TLR for 11 endovascular interventions.^10^^,^^12^^,^^13^^,^^21^, ^22^, ^23^, ^24^, ^25^, ^26^, ^27^, ^28^, ^29^, ^30^, ^31^^,^^33^^,^^35^, ^36^, ^37^, ^38^^,^^41^, ^42^, ^43^^,^^46^, ^47^, ^48^, ^49^, ^50^, ^51^, ^52^^,^^54^, ^55^, ^56^, ^57^, ^58^, ^59^, ^60^, ^61^, ^62^, ^63^, ^64^, ^65^, ^66^, ^67^, ^68^, ^69^, ^70^, ^71^, ^72^, ^73^ There was significant heterogeneity across trials (*I*^2^ = 44.5%; 95% CI, 18.9%-62.0%). A+DCB (OR, 0.13), BMS (OR, 0.46), brachy (OR, 0.38), CSG (OR, 0.27), DCB (OR, 0.27), DCB+BMS (OR, 0.23), and DES (OR, 0.39) were superior to PTA. BMS (OR, 1.71) was inferior to DCB (Figure 4B). When ranked, A+DCB had the highest P-score (0.89) (Table 3).

A total of 18 studies including 2885 patients assessed improvement in Rutherford classification at 12 months for 7 endovascular interventions.^25^^,^^26^^,^^30^^,^^31^^,^^35^^,^^36^^,^^40^^,^^47^^,^^49^^,^^50^^,^^57^^,^^58^^,^^60^^,^^61^^,^^63^^,^^65^^,^^67^^,^^70^ There was no significant heterogeneity across trials (*I*^2^ = 26.2%; 95% CI, 0.0%-62.6%). BMS (OR, 2.82), DCB (OR, 1.91), DCB+BMS (OR, 15.08), and DES (OR, 3.72) all significantly improved Rutherford classification compared with PTA. DCB+BMS was superior to A+DCB (OR, 0.05), BMS (OR, 0.19), brachy (OR, 0.06), and DCB (OR, 0.13) (Figure 4C). When ranked, DCB+BMS had the highest P-score (0.99) (Table 3).

A limited number of trials assessed 12-month changes in EuroQol-5 Dimension and Walking Impairment Questionnaire scores. Although point estimates favored DCB over PTA, they were not significant.

### Meta-regression analysis

Based on meta-regression, trials comparing DCB vs PTA with a higher proportion of de novo lesions (*P* < .0001), female patients (*P* = .04), and severely calcified lesions (*P* = .03) had greater effect size for DCBs improving Rutherford category. Trials with a higher proportion of de novo lesions (*P* = .014) had greater effect size favoring DCBs for 12-month TLR. Trials with a higher proportion of chronic total occlusions (*P* = .008) had greater 12-month mortality in the DCB arms (Supplementary Table S5). When accounting for multiple testing, only effect modification of de novo lesions remained significant.

## Discussion

This network meta-analysis, based on rigorously conducted contemporary RCTs with independent and core laboratory adjudication of end points, provides up to date, comprehensive and clinically relevant insights into the relative safety and efficacy of the numerous endovascular devices available to treat SFA disease in primarily IC patients. Technical success was highest for CSG, BMS, and A+DCB, and 12-month primary patency was superior for virtually all newer endovascular interventions compared with PTA (Central Illustration). Therapies with improved primary patency compared to PTA were also associated with more frequent improvement in symptoms, with DCB+BMS being particularly highly ranked for 12-month improvement in Rutherford classification. The 12-month mortality and major amputation rates were similar between treatments, although these comparisons were limited by the small number of studies included and low event rates (considering most were IC patients who generally have lower rates of these events than CLTI patients). Comparisons of changes in Walking Impairment Questionnaire and EuroQol-5 Dimension were also limited by number of studies, although their point estimates favored DCB over PTA.

**Central Illustration:**
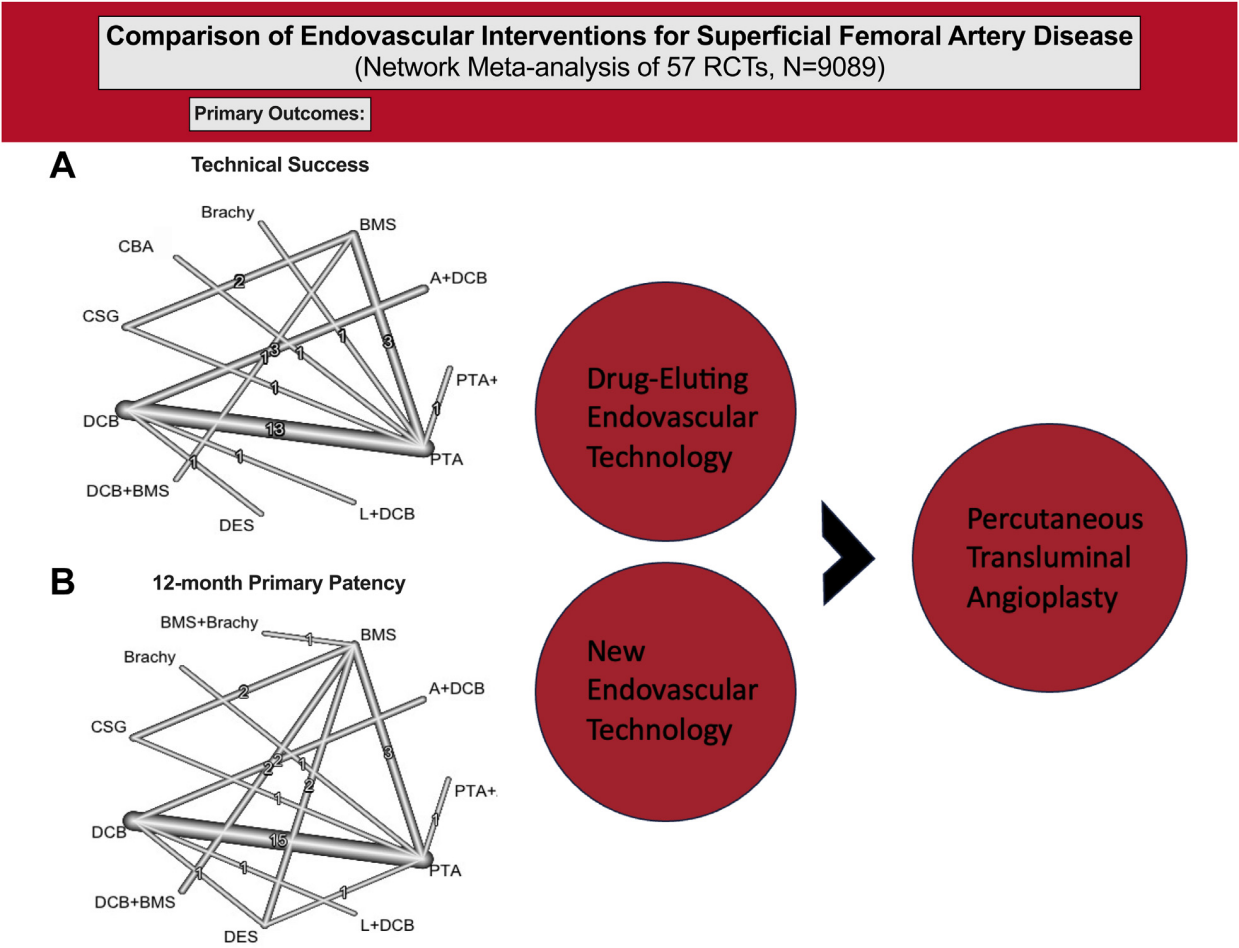
Primary end points network plots of (A) technical success and (B) 12-month primary patency. A, atherectomy; BMS, bare metal stent; brachy, brachytherapy; CBA, cutting balloon angioplasty; CSG, covered stent graft; DCB, drug-coated balloon; DES, drug-eluting stent; L, lithotripsy; PTA, percutaneous transluminal angioplasty; RCT, randomized controlled trial.

Our results are consistent with findings of previous meta-analyses that demonstrate that PTA alone for SFA lesions is inferior to other endovascular options in primary patency and technical success.^74^^,^^75^ Our meta-analysis also confirmed that these findings were consistent among trials including patients with in-stent restenosis, which was not previously evaluated. It also showed that PTA was inferior to other interventions, including DCBs, in symptom improvement. This is not surprising because negative remodeling and neointimal hyperplasia following PTA are mitigated by therapies such as stents and DCBs.^76^, ^77^, ^78^ This adds to the growing evidence that a PTA-alone strategy for SFA disease is insufficient^79^ for most lesions despite it being used in up to 30% of cases.^5^^,^^80^ Although PTA alone may have been overused in the past due to the now discredited link between paclitaxel and increased mortality,^81^, ^82^, ^83^ these results highlight the benefit of drug-eluting therapy.

This study addresses, in part, the gap in knowledge regarding device selection in femoropopliteal disease identified by previous guidelines.^9^^,^^15^ Both documents give relatively little guidance on device selection and only give weak recommendations for newer therapies relative to PTA and only when PTA gives a suboptimal result. Our study shows that technical success is superior with the use of newer endovascular therapies as a first-line strategy. Additionally, 12-month primary patency and even more importantly, patient symptoms, are also improved when newer endovascular therapies are used as a first-line strategy. As drug-eluting therapies including DCBs continue to become standard of care for these patients, the comparisons presented here using DCB as the comparator will provide valuable insight for device selection in modern practice.

Highly calcified SFA lesions are difficult to treat due to decreased vessel compliance, increased risk of dissection with balloon expansion, and decreased delivery of drug therapy.^84^ Currently, the mainstay therapy for highly calcified lesions includes vessel preparation strategies with intravascular L and A, often followed by PTA or DCB.^30^^,^^85^^,^^86^ Our analysis suggests both are viable options, particularly when paired with DCBs, given that L+DCB and A+DCB were highly ranked for 12-month primary patency. However, given the limited number of studies including these interventions, this result should be interpreted with caution.

Subgroup analyses by meta-regression suggested that patients with de novo lesions had greater benefit from DCB for symptom improvement and TLR compared with those with in-stent restenosis. It is well established that DCBs are superior to PTA alone in patients with in-stent restenosis^49^^,^^50^^,^^52^; however, this study demonstrates that the benefit is less strong than in de novo lesions. There is abundant evidence that DCBs are superior for heavily calcified lesions^59^^,^^87^ and for women,^88^ which are both supported by our meta-regression analysis as well. Although our meta-regression analysis suggested there may be increased mortality with DCBs in trials that enrolled more patients with total occlusions, this does not have a physiologic basis and, as mentioned above, this association has been repeatedly debunked. More likely this finding was the result of type I error from multiple testing as evidenced by all meta-regressions, besides those looking at de novo lesions, no longer being significant after adjusting *P* values for multiple testing.

### Limitations

This study has several limitations. First, this is a post hoc analysis of trial-level data; therefore, these results are only hypothesis-generating. For many comparisons there was significant heterogeneity across trials, likely reflecting the varying patient/lesion selection in the trials for different devices, which may affect the interpretability of the results, particularly for interventions used in vastly different patient populations. However, in most cases, heterogeneity was at most moderate^89^ and was explained by differences in baseline characteristics identified by meta-regression including lesion calcification and in-stent restenosis lesions. Additionally, many outcomes and interventions had few, if any, studies contributing to the network, limiting many comparisons and making the results susceptible to selection bias based on which studies reported which outcomes. Finally, the cohort had a male predominance, which limits the generalizability of the results to the broader population, although this is a limitation in trial enrollment and not one specifically of this meta-analysis.

## Conclusion

This network meta-analysis supports the benefit of new endovascular therapies, including drug-eluting therapy, for the treatment of SFA disease compared with PTA alone. Future studies focused on device selection in SFA disease are warranted.

## References

[1] Nehler M.R., Duval S., Diao L. (2014). Epidemiology of peripheral arterial disease and critical limb ischemia in an insured national population. J Vasc Surg.

[2] Mahoney E.M., Wang K., Keo H.H. (2010). Vascular hospitalization rates and costs in patients with peripheral artery disease in the United States. Circ Cardiovasc Qual Outcomes.

[3] Conte M.S., Bradbury A.W., Kolh P. (2019). Global vascular guidelines on the management of chronic limb-threatening ischemia. Eur J Vasc Endovasc Surg.

[4] Poredos P., Cevc M., Blinc A. (2021). Characteristics of atherosclerosis in femoropopliteal artery and its clinical relevance. Atherosclerosis.

[5] Farber A., Menard M.T., Conte M.S. (2022). Surgery or endovascular therapy for chronic limb-threatening ischemia. N Engl J Med.

[6] Bradbury A.W., Moakes C.A., Popplewell M. (2023). A vein bypass first versus a best endovascular treatment first revascularisation strategy for patients with chronic limb threatening ischaemia who required an infra-popliteal, with or without an additional more proximal infra-inguinal revascularisation procedure to restore limb perfusion (BASIL-2): an open-label, randomised, multicentre, phase 3 trial. Lancet.

[7] Primary Panel, Abramson B.L., Al-Omran M. (2022). Canadian Cardiovascular Society 2022 guidelines for peripheral arterial disease. Can J Cardiol.

[8] Foley K.M., Kennedy K.F., Lima F.V. (2024). Treatment variability among patients hospitalized for chronic limb-threatening ischemia: an analysis of the 2016 to 2018 US National Inpatient Sample. J Am Heart Assoc.

[9] Gornik H.L., Aronow H.D., Goodney P.P. (2024). 2024 ACC/AHA/AACVPR/APMA/ABC/SCAI/SVM/SVN/SVS/SIR/VESS guideline for the management of lower extremity peripheral artery disease: a report of the American College of Cardiology/American Heart Association joint committee on clinical practice guidelines. Circulation.

[10] Tepe G., Brodmann M., Bachinsky W. (2022). Intravascular lithotripsy for peripheral artery calcification: mid-term outcomes from the randomized disrupt PAD III trial. J Soc Cardiovasc Angiogr Interv.

[11] Bosiers M., Deloose K., Callaert J. (2020). Stent-grafts are the best way to treat complex in-stent restenosis lesions in the superficial femoral artery: 24-month results from a multicenter randomized trial. J Cardiovasc Surg (Torino).

[12] Liistro F., Angioli P., Porto I. (2019). Drug-eluting balloon versus drug-eluting stent for complex femoropopliteal arterial lesions: the DRASTICO study. J Am Coll Cardiol.

[13] Gouëffic Y., Sauguet A., Desgranges P. (2020). A polymer-free paclitaxel-eluting stent versus a bare-metal stent for de novo femoropopliteal lesions: the BATTLE trial. J Am Coll Cardiol Intv.

[14] Writing Committee Members, Gornik H.L., Aronow H.D. (2024). 2024 ACC/AHA/AACVPR/APMA/ABC/SCAI/SVM/SVN/SVS/SIR/VESS guideline for the management of lower extremity peripheral artery disease: a report of the American College of Cardiology/American Heart Association joint committee on clinical practice guidelines. J Am Coll Cardiol.

[15] Nordanstig J., Behrendt C.A., Baumgartner I. (2024). Editor’s Choice--European Society for Vascular Surgery (ESVS) 2024 clinical practice guidelines on the management of asymptomatic lower limb peripheral arterial disease and intermittent claudication. Eur J Vasc Endovasc Surg.

[16] Page M.J., McKenzie J.E., Bossuyt P.M. (2021). The PRISMA 2020 statement: an updated guideline for reporting systematic reviews. Int J Surg.

[17] Rucker G., Schwarzer G. (2015). Ranking treatments in frequentist network meta-analysis works without resampling methods. BMC Med Res Methodol.

[18] Sterne J.A.C., Savović J., Page M.J. (2019). RoB 2: a revised tool for assessing risk of bias in randomised trials. BMJ.

[19] Therasse E., Donath D., Elkouri S. (2016). Results of a randomized clinical trial of external beam radiation to prevent restenosis after superficial femoral artery stenting. J Vasc Surg.

[20] Wolfram R.M., Budinsky A.C., Pokrajac B., Pötter R., Minar E. (2005). Vascular brachytherapy with 192Ir after femoropopliteal stent implantation in high-risk patients: twelve-month follow-up results from the Vienna-5 trial. Radiology.

[21] Geraghty P.J., Mewissen M.W., Jaff M.R., Ansel G.M. (2013). Three-year results of the VIBRANT trial of VIABAHN endoprosthesis versus bare nitinol stent implantation for complex superficial femoral artery occlusive disease. J Vasc Surg.

[22] Lammer J., Zeller T., Hausegger K.A. (2013). Heparin-bonded covered stents versus bare-metal stents for complex femoropopliteal artery lesions: the randomized VIASTAR trial (Viabahn endoprosthesis with PROPATEN bioactive surface [VIA] versus bare nitinol stent in the treatment of long lesions in superficial femoral artery occlusive disease). J Am Coll Cardiol.

[23] de Boer S.W., van den Heuvel D.A.F., de Vries-Werson D.A.B. (2017). Short-term results of the RAPID randomized trial of the Legflow paclitaxel-eluting balloon with Supera stenting vs Supera stenting alone for the treatment of intermediate and long superficial femoral artery lesions. J Endovasc Ther.

[24] Liistro F., Grotti S., Porto I. (2013). Drug-eluting balloon in peripheral intervention for the superficial femoral artery: the DEBATE-SFA randomized trial (drug eluting balloon in peripheral intervention for the superficial femoral artery). J Am Coll Cardiol Intv.

[25] Tacke J., Müller-Hülsbeck S., Schröder H. (2019). The randomized Freeway stent study: drug-eluting balloons outperform standard balloon angioplasty for postdilatation of nitinol stents in the SFA and PI segment. Cardiovasc Intervent Radiol.

[26] Gouëffic Y., Torsello G., Zeller T. (2022). Efficacy of a drug-eluting stent versus bare metal stents for symptomatic femoropopliteal peripheral artery disease: primary results of the EMINENT randomized trial. Circulation.

[27] Duda S.H., Bosiers M., Lammer J. (2006). Drug-eluting and bare nitinol stents for the treatment of atherosclerotic lesions in the superficial femoral artery: long-term results from the SIROCCO trial. J Endovasc Ther.

[28] Cai Z., Guo L., Qi L. (2020). Midterm outcome of directional atherectomy combined with drug-coated balloon angioplasty versus drug-coated balloon angioplasty alone for femoropopliteal arteriosclerosis obliterans. Ann Vasc Surg.

[29] Shammas N.W., Purushottam B., Shammas W.J. (2022). Jetstream atherectomy followed by paclitaxel-coated balloons versus balloon angioplasty followed by paclitaxel-coated balloons: twelve-month exploratory results of the prospective randomized JET-RANGER study. Vasc Health Risk Manag.

[30] Zeller T., Langhoff R., Rocha-Singh K.J. (2017). Directional atherectomy followed by a paclitaxel-coated balloon to inhibit restenosis and maintain vessel patency: twelve-month results of the DEFINITIVE AR study. Circ Cardiovasc Interv.

[31] Bausback Y., Wittig T., Schmidt A. (2019). Drug-eluting stent versus drug-coated balloon revascularization in patients with femoropopliteal arterial disease. J Am Coll Cardiol.

[32] Becquemin J.P., Favre J.P., Marzelle J., Nemoz C., Corsin C., Leizorovicz A. (2003). Systematic versus selective stent placement after superficial femoral artery balloon angioplasty: a multicenter prospective randomized study. J Vasc Surg.

[33] Chalmers N., Walker P.T., Belli A.M. (2013). Randomized trial of the SMART stent versus balloon angioplasty in long superficial femoral artery lesions: the SUPER study. Cardiovasc Intervent Radiol.

[34] Dick P., Wallner H., Sabeti S. (2009). Balloon angioplasty versus stenting with nitinol stents in intermediate length superficial femoral artery lesions. Catheter Cardiovasc Interv.

[35] Krankenberg H., Schlüter M., Steinkamp H.J. (2007). Nitinol stent implantation versus percutaneous transluminal angioplasty in superficial femoral artery lesions up to 10 cm in length: the femoral artery stenting trial (FAST). Circulation.

[36] Laird J.R., Katzen B.T., Scheinert D. (2010). Nitinol stent implantation versus balloon angioplasty for lesions in the superficial femoral artery and proximal popliteal artery: twelve-month results from the RESILIENT randomized trial. Circ Cardiovasc Interv.

[37] Schillinger M., Sabeti S., Loewe C. (2006). Balloon angioplasty versus implantation of nitinol stents in the superficial femoral artery. N Engl J Med.

[38] Iida O., Urasawa K., Komura Y. (2019). Self-expanding nitinol stent vs percutaneous transluminal angioplasty in the treatment of femoropopliteal lesions: 3-year data from the SM-01 trial. J Endovasc Ther.

[39] Schillinger M., Sabeti S., Dick P. (2007). Sustained benefit at 2 years of primary femoropopliteal stenting compared with balloon angioplasty with optional stenting. Circulation.

[40] Diehm N., Silvestro A., Do D.D. (2005). Endovascular brachytherapy after femoropopliteal balloon angioplasty fails to show robust clinical benefit over time. J Endovasc Ther.

[41] Krueger K., Zaehringer M., Bendel M. (2004). De novo femoropopliteal stenoses: endovascular gamma irradiation following angioplasty—angiographic and clinical follow-up in a prospective randomized controlled trial. Radiology.

[42] Minar E., Pokrajac B., Maca T. (2000). Endovascular brachytherapy for prophylaxis of restenosis after femoropopliteal angioplasty: results of a prospective randomized study. Circulation.

[43] Pokrajac B., Pötter R., Wolfram R.M. (2005). Endovascular brachytherapy prevents restenosis after femoropopliteal angioplasty: results of the Vienna-3 randomised multicenter study. Radiother Oncol.

[44] Therasse E., Donath D., Lespérance J. (2005). External beam radiation to prevent restenosis after superficial femoral artery balloon angioplasty. Circulation.

[45] Amighi J., Schillinger M., Dick P. (2008). De novo superficial femoropopliteal artery lesions: peripheral cutting balloon angioplasty and restenosis rates--randomized controlled trial. Radiology.

[46] Poncyljusz W., Falkowski A., Safranow K., Rać M., Zawierucha D. (2013). Cutting-balloon angioplasty versus balloon angioplasty as treatment for short atherosclerotic lesions in the superficial femoral artery: randomized controlled trial. Cardiovasc Intervent Radiol.

[47] Bosiers M., Deloose K., Callaert J. (2015). Superiority of stent-grafts for in-stent restenosis in the superficial femoral artery: twelve-month results from a multicenter randomized trial. J Endovasc Ther.

[48] Fanelli F., Cannavale A., Corona M., Lucatelli P., Wlderk A., Salvatori F.M. (2014). The “DEBELLUM”--lower limb multilevel treatment with drug eluting balloon--randomized trial: 1-year results. J Cardiovasc Surg (Torino).

[49] Krankenberg H., Tübler T., Ingwersen M. (2015). Drug-coated balloon versus standard balloon for superficial femoral artery in-stent restenosis: the randomized Femoral Artery In-Stent Restenosis (FAIR) Trial. Circulation.

[50] Liao C.J., Song S.H., Li T., Zhang Y., Zhang W.D. (2019). Randomized controlled trial of orchid drug-coated balloon versus standard percutaneous transluminal angioplasty for treatment of femoropopliteal artery in-stent restenosis. Int Angiol.

[51] Iida O., Soga Y., Urasawa K. (2018). Drug-coated balloon vs standard percutaneous transluminal angioplasty for the treatment of atherosclerotic lesions in the superficial femoral and proximal popliteal arteries: one-year results of the MDT-2113 SFA Japan randomized trial. J Endovasc Ther.

[52] Ott I., Cassese S., Groha P. (2017). ISAR-PEBIS (paclitaxel-eluting balloon versus conventional balloon angioplasty for in-stent restenosis of superficial femoral artery): a randomized trial. J Am Heart Assoc.

[53] Wyttenbach R., Corti R., Alerci M. (2007). Effects of percutaneous transluminal angioplasty and endovascular brachytherapy on vascular remodeling of human femoropopliteal artery: 2 years follow-up by noninvasive magnetic resonance imaging. Eur J Vasc Endovasc Surg.

[54] Tepe G., Zeller T., Albrecht T. (2008). Local delivery of paclitaxel to inhibit restenosis during angioplasty of the leg. N Engl J Med.

[55] Werk M., Langner S., Reinkensmeier B. (2008). Inhibition of restenosis in femoropopliteal arteries: paclitaxel-coated versus uncoated balloon: femoral paclitaxel randomized pilot trial. Circulation.

[56] Buszman P.P., Nowakowski P., Milewski K. (2018). Clinical randomized trial evaluating novel, microcrystalline, and biocompatible polymer paclitaxel-coated balloon for the treatment of femoropopliteal occlusive disease: the BIOPAC trial. J Am Coll Cardiol Intv.

[57] Jia X., Zhang J., Zhuang B. (2016). Acotec drug-coated balloon catheter: randomized, multicenter, controlled clinical study in femoropopliteal arteries: evidence from the AcoArt I trial. J Am Coll Cardiol Intv.

[58] Kinstner C.M., Lammer J., Willfort-Ehringer A. (2016). Paclitaxel-eluting balloon versus standard balloon angioplasty in in-stent restenosis of the superficial femoral and proximal popliteal artery: 1-year results of the PACUBA trial. J Am Coll Cardiol Intv.

[59] Krishnan P., Faries P., Niazi K. (2017). Stellarex drug-coated balloon for treatment of femoropopliteal disease: twelve-month outcomes from the randomized ILLUMENATE pivotal and pharmacokinetic studies. Circulation.

[60] Sachar R., Soga Y., Ansari M.M. (2021). 1-year results from the RANGER II SFA randomized trial of the Ranger drug-coated balloon. J Am Coll Cardiol Intv.

[61] Scheinert D., Schulte K.L., Zeller T., Lammer J., Tepe G. (2015). Paclitaxel-releasing balloon in femoropopliteal lesions using a BTHC excipient: twelve-month results from the BIOLUX P-I randomized trial. J Endovasc Ther.

[62] Scheinert D., Duda S., Zeller T. (2014). The LEVANT I (Lutonix paclitaxel-coated balloon for the prevention of femoropopliteal restenosis) trial for femoropopliteal revascularization: first-in-human randomized trial of low-dose drug-coated balloon versus uncoated balloon angioplasty. J Am Coll Cardiol Intv.

[63] Schroeder H., Werner M., Meyer D.R. (2017). Low-dose paclitaxel-coated versus uncoated percutaneous transluminal balloon angioplasty for femoropopliteal peripheral artery disease: one-year results of the ILLUMENATE European randomized clinical trial (randomized trial of a novel paclitaxel-coated percutaneous angioplasty balloon). Circulation.

[64] Steiner S., Willfort-Ehringer A., Sievert H. (2018). 12-Month results from the first-in-human randomized study of the Ranger paclitaxel-coated balloon for femoropopliteal treatment. J Am Coll Cardiol Intv.

[65] Teichgräber U., Lehmann T., Aschenbach R. (2020). Efficacy and safety of a novel paclitaxel-nano-coated balloon for femoropopliteal angioplasty: one-year results of the EffPac trial. EuroIntervention.

[66] Tepe G., Gögebakan Ö., Redlich U. (2017). Angiographic and clinical outcomes after treatment of femoro-popliteal lesions with a novel paclitaxel-matrix-coated balloon catheter. Cardiovasc Intervent Radiol.

[67] Tepe G., Schroeder H., Albrecht T. (2020). Paclitaxel-coated balloon vs uncoated balloon angioplasty for treatment of in-stent restenosis in the superficial femoral and popliteal arteries: the COPA CABANA trial. J Endovasc Ther.

[68] Tepe G., Laird J., Schneider P. (2015). Drug-coated balloon versus standard percutaneous transluminal angioplasty for the treatment of superficial femoral and popliteal peripheral artery disease: 12-month results from the IN.PACT SFA randomized trial. Circulation.

[69] Werk M., Albrecht T., Meyer D.R. (2012). Paclitaxel-coated balloons reduce restenosis after femoro-popliteal angioplasty: evidence from the randomized PACIFIER trial. Circ Cardiovasc Interv.

[70] Ye W., Zhang X., Dai X. (2021). Reewarm™ PTX drug-coated balloon in the treatment of femoropopliteal artery disease: a multi-center, randomized controlled trial in China. Int J Cardiol.

[71] Dake M.D., Ansel G.M., Jaff M.R. (2011). Paclitaxel-eluting stents show superiority to balloon angioplasty and bare metal stents in femoropopliteal disease: twelve-month Zilver PTX randomized study results. Circ Cardiovasc Interv.

[72] Dippel E.J., Makam P., Kovach R. (2015). Randomized controlled study of excimer laser atherectomy for treatment of femoropopliteal in-stent restenosis: initial results from the EXCITE ISR trial (EXCImer Laser Randomized Controlled Study for Treatment of FemoropopliTEal In-Stent Restenosis). J Am Coll Cardiol Intv.

[73] Rosenfield K., Jaff M.R., White C.J. (2015). Trial of a paclitaxel-coated balloon for femoropopliteal artery disease. N Engl J Med.

[74] Antonopoulos C.N., Mylonas S.N., Moulakakis K.G. (2017). A network meta-analysis of randomized controlled trials comparing treatment modalities for de novo superficial femoral artery occlusive lesions. J Vasc Surg.

[75] Zhou Y., Zhang Z., Lin S. (2020). Comparative effectiveness of endovascular treatment modalities for de novo femoropopliteal lesions: a network meta-analysis of randomized controlled trials. J Endovasc Ther.

[76] Gray W.A., Granada J.F. (2010). Drug-coated balloons for the prevention of vascular restenosis. Circulation.

[77] Joner M., Finn A.V., Farb A. (2006). Pathology of drug-eluting stents in humans: delayed healing and late thrombotic risk. J Am Coll Cardiol.

[78] Rozenman Y., Gilon D., Welber S., Sapoznikov D., Gotsman M.S. (1993). Clinical and angiographic predictors of immediate recoil after successful coronary angioplasty and relation to late restenosis. Am J Cardiol.

[79] Sridharan N.D., Boitet A., Smith K. (2018). Cost-effectiveness analysis of drug-coated therapies in the superficial femoral artery. J Vasc Surg.

[80] Bertges D.J., White R., Cheng Y.C. (2021). Registry Assessment of Peripheral Interventional Devices objective performance goals for superficial femoral and popliteal artery peripheral vascular interventions. J Vasc Surg.

[81] Laird J.A., Schneider P.A., Jaff M.R. (2019). Long-term clinical effectiveness of a drug-coated balloon for the treatment of femoropopliteal lesions. Circ Cardiovasc Interv.

[82] Lyden S.P., Faries P.L., Niazi K.A.K. (2022). No mortality signal with Stellarex low-dose paclitaxel DCB: ILLUMENATE pivotal 4-year outcomes. J Endovasc Ther.

[83] Dake M.D., Ansel G.M., Bosiers M. (2020). Paclitaxel-coated Zilver PTX drug-eluting stent treatment does not result in increased long-term all-cause mortality compared to uncoated devices. Cardiovasc Intervent Radiol.

[84] Rocha-Singh K.J., Zeller T., Jaff M.R. (2014). Peripheral arterial calcification: prevalence, mechanism, detection, and clinical implications. Catheter Cardiovasc Interv.

[85] Nasiri A., Kim H., Gurusamy V., Benenati J.F. (2022). Management of calcification: rational and technical considerations for intravascular lithotripsy. Tech Vasc Interv Radiol.

[86] Fanelli F., Cannavale A., Gazzetti M. (2014). Calcium burden assessment and impact on drug-eluting balloons in peripheral arterial disease. Cardiovasc Intervent Radiol.

[87] Caradu C., Lakhlifi E., Colacchio E.C. (2019). Systematic review and updated meta-analysis of the use of drug-coated balloon angioplasty versus plain old balloon angioplasty for femoropopliteal arterial disease. J Vasc Surg.

[88] Krishnan P., Tarricone A., Purushottam B. (2020). Gender differences in the outcomes of drug-coated balloon treatment in symptomatic femoropopliteal arterial disease. Vasc Endovascular Surg.

[89] Alba A.C., Alexander P.E., Chang J., MacIsaac J., DeFry S., Guyatt G.H. (2016). High statistical heterogeneity is more frequent in meta-analysis of continuous than binary outcomes. J Clin Epidemiol.

