# Supplementary material for: Comparison of Endovascular Interventions for the Treatment of Superficial Femoral Artery Disease: A Network Meta-analysis
Source: J Soc Cardiovasc Angiogr Interv. 2025 Jan 21;4(1):102432. doi: 10.1016/j.jscai.2024.102432 (PMC11887560; doi:10.1016/j.jscai.2024.102432)
Supplement: Supplementary Figures Tables [file mmc1.docx]

**Supplemental Material**

**Supplemental Figure S1. Funnel plots for primary outcomes.**

(A) Technical success; (B) primary patency

**Supplemental Figure S2. Funnel plots for secondary outcomes**.

(A) All-cause death at 12 months; (B) target lesion revascularization at 12 months; (C) primary patency at 6 months; (D) improvement in Rutherford category at 12 months; (E) major amputation at 12 months; (F) EQ-5D score change at 12 months.

**Supplemental Figure S3. Secondary outcomes of the network meta-analysis.**

(A) 6-month primary patency and (B) 12-month major amputation. Endovascular interventions with their comparators are on the left of the y-axis, with corresponding odds ratio (OR) with 95% confidence intervals (CI) displayed on the right. A+DCB, atherectomy + drug-coated balloon; BMS, bare metal stent; BMS+brachy, bare metal stent + brachytherapy; Brachy, brachytherapy; CBA, cutting balloon angioplasty; CSG, covered stent graft; DCB, drug-coated balloon; DCB+BMS, drug-coated balloon + bare metal stent; DES, drug-eluting stent; L+DCB, lithotripsy + drug-coated balloon; PTA, percutaneous transluminal angioplasty; PTA+A, percutaneous transluminal angioplasty + atherectomy.

**Supplemental Figure S4. Network plots for secondary safety and efficacy outcomes.**

Line thickness is weight by amount of randomized controlled trials used to compare the 2 groups by outcome. (A) All-cause death at 12 months; (B) target lesion revascularization at 12 months; (C) primary patency at 6 months; (D) improvement in Rutherford category at 12 months; (E) major amputation at 12 months; (F) EQ-5D score change at 12 months.

**Supplemental Table S1. Trial level details of studies included in the network meta-analysis.** “1” denotes use of trial in network meta-analysis, “0” denotes absence of trial from the network.

| Author with Trial Title | Comparisons | Trial Start (month-year) | Trial Enrollment End (month-year) | Sample size (control) | Sample Size (experimental) | Published (year) | Primary Patency | Technical Success | Major Amputation | Primary patency 6 | Rutherford 12 | TLR 12 months | All cause death at 12 months | WIQ Score Change 12 months | EQ-5D Score Change at 12 months |
| --- | --- | --- | --- | --- | --- | --- | --- | --- | --- | --- | --- | --- | --- | --- | --- |
| Therasse et al. | BMS vs. BMS+Brachy | Nov-06 | Dec-11 | 78 | 77 | 2016 | 0 | 0 | 0 | 0 | 0 | 0 | 0 | 0 | 0 |
| Wolfram et al. (Vienna-5 trial) | BMS vs. BMS+Brachy | Jun-99 | Jan-02 | 46 | 42 | 2005 | 1 | 0 | 0 | 1 | 0 | 0 | 0 | 0 | 0 |
| Geraghty et al. (VIBRANT) | BMS vs. CSG | Oct-05 | Dec-07 | 76 | 72 | 2013 | 1 | 1 | 0 | 1 | 0 | 1 | 0 | 0 | 0 |
| Lammer et al. (VIASTAR) | BMS vs. CSG | Mar-09 | Mar-11 | 69 | 71 | 2013 | 1 | 1 | 0 | 0 | 0 | 1 | 0 | 0 | 0 |
| de Boer et al. (RAPID) | BMS vs. DCB+BMS | Jun-12 | May-16 | 80 | 80 | 2017 | 1 | 1 | 1 | 1 | 0 | 1 | 1 | 0 | 0 |
| Liistro et al. (DEBATE-SFA trial) | BMS vs. DCB+BMS | Nov-10 | Nov-11 | 51 | 53 | 2013 | 0 | 1 | 1 | 0 | 0 | 1 | 1 | 0 | 0 |
| Tacke et al. (FREEWAY trial) | BMS vs. DCB+BMS | NA | 2015 | 99 | 105 | 2019 | 1 | 0 | 1 | 1 | 1 | 1 | 1 | 0 | 0 |
| Goueffic et al. (EMIMENT trial) | BMS vs. DES | Oct-16 | Mar-20 | 267 | 508 | 2022 | 1 | 0 | 1 | 0 | 1 | 1 | 1 | 0 | 0 |
| Duda et al. (SIROCCO trial) | BMS vs. DES | Aug-22 | Dec-02 | 46 | 47 | 2006 | 0 | 0 | 1 | 0 | 0 | 1 | 0 | 0 | 0 |
| Goueffic et al. (BATTLE trial) | BMS vs. DES | Mar-14 | Aug-16 | 85 | 86 | 2020 | 1 | 1 | 1 | 1 | 0 | 1 | 0 | 0 | 1 |
| Cai et al. | DCB vs. A+DCB | Jun-16 | Jun-18 | 49 | 45 | 2020 | 1 | 1 | 1 | 1 | 0 | 1 | 1 | 0 | 0 |
| Shammas et al. (JET-RANGER trial) | DCB vs. A+DCB | Mar-18 | Apr-20 | 16 | 31 | 2022 | 0 | 1 | 1 | 1 | 0 | 1 | 1 | 0 | 0 |
| Zeller et al. (DEFINITIVE AR trial) | DCB vs. A+DCB | Aug-11 | May-13 | 54 | 48 | 2017 | 1 | 1 | 1 | 1 | 1 | 1 | 1 | 0 | 1 |
| Bausback et al. | DCB vs. DES | Mar-12 | May-14 | 75 | 75 | 2019 | 1 | 1 | 1 | 1 | 1 | 1 | 1 | 0 | 0 |
| Liistro et al. (DRASTICO trial) | DCB vs. DES | Jan-13 | Jun-17 | 96 | 96 | 2019 | 0 | 1 | 1 | 0 | 0 | 1 | 1 | 0 | 0 |
| Tepe et al. (Disrupt PAD III trial) | DCB vs. L+DCB | Feb-17 | Feb-20 | 153 | 153 | 2022 | 1 | 1 | 0 | 0 | 0 | 1 | 0 | 0 | 0 |
| Becquemin et al. | PTA vs. BMS | Apr-95 | Dec-97 | 112 | 115 | 2003 | 0 | 0 | 0 | 0 | 0 | 0 | 1 | 0 | 0 |
| Chalmers et al. (SUPER trial) | PTA vs. BMS | Apr-05 | Dec-06 | 76 | 74 | 2013 | 1 | 1 | 1 | 0 | 0 | 1 | 1 | 0 | 1 |
| Dick et al. | PTA vs. BMS | NA | NA | 39 | 34 | 2009 | 0 | 0 | 0 | 0 | 0 | 0 | 0 | 0 | 0 |
| Krankenburg et al. (FAST trial) | PTA vs. BMS | Jan-04 | Mar-05 | 121 | 123 | 2007 | 0 | 0 | 0 | 0 | 1 | 1 | 1 | 0 | 0 |
| Laird et al. (RESILIENT trial) | PTA vs. BMS | Dec-04 | Aug-06 | 72 | 134 | 2010 | 1 | 1 | 0 | 1 | 1 | 1 | 1 | 0 | 0 |
| lida et al. (SM-01 trial) | PTA vs. BMS | Jul-10 | Aug-14 | 52 | 51 | 2019 | 1 | 1 | 0 | 1 | 0 | 1 | 0 | 0 | 0 |
| Schillinger et al. | PTA vs. BMS | Apr-03 | Aug-04 | 53 | 51 | 2006 | 0 | 0 | 1 | 0 | 0 | 1 | 1 | 0 | 0 |
| Diehm et al. | PTA vs. Brachy | 1997 | 2002 | 75 | 72 | 2005 | 0 | 1 | 1 | 0 | 1 | 0 | 0 | 0 | 0 |
| Krueger et al. | PTA vs. Brachy | NA | NA | 15 | 15 | 2004 | 0 | 0 | 0 | 0 | 0 | 1 | 0 | 0 | 0 |
| Minar et al. | PTA vs. Brachy | Nov-96 | Aug-98 | 56 | 57 | 2000 | 0 | 0 | 0 | 1 | 0 | 1 | 1 | 0 | 0 |
| Pokrajac et al. (Vienna-3) | PTA vs. Brachy | Oct-98 | Jul-01 | 46 | 50 | 2005 | 1 | 1 | 0 | 1 | 0 | 1 | 0 | 0 | 0 |
| Therasse et al. | PTA vs. Brachy | Jul-98 | Mar-02 | 24 | 25 | 2005 | 0 | 0 | 0 | 0 | 0 | 0 | 0 | 0 | 0 |
| Amighi et al. | PTA vs. CBA | Aug-04 | Jun-06 | 22 | 21 | 2008 | 0 | 0 | 0 | 0 | 0 | 0 | 0 | 0 | 0 |
| Poncylijusz et al. | PTA vs. CBA | Jun-07 | Dec-09 | 30 | 30 | 2013 | 0 | 1 | 1 | 0 | 0 | 1 | 0 | 0 | 0 |
| Bosiers et al. | PTA vs. CSG | Jun-10 | Feb-12 | 44 | 39 | 2015 | 1 | 1 | 0 | 1 | 1 | 1 | 1 | 0 | 0 |
| Fanelli et al. (DEBELLUM trial) | PTA vs. DCB | Sep-10 | Mar-11 | 25 | 25 | 2014 | 1 | 1 | 0 | 1 | 0 | 1 | 1 | 0 | 0 |
| Krankenburg et al. (FAIR trial) | PTA vs. DCB | Jan-10 | Dec-12 | 57 | 62 | 2015 | 0 | 1 | 1 | 0 | 1 | 1 | 1 | 0 | 0 |
| Liao et al. | PTA vs. DCB | NA | NA | 36 | 38 | 2019 | 1 | 1 | 1 | 0 | 1 | 1 | 1 | 0 | 1 |
| lida et al. (MDT-2113 SFA trial) | PTA vs. DCB | Sep-13 | Apr-18 | 32 | 68 | 2018 | 1 | 1 | 1 | 1 | 0 | 1 | 1 | 0 | 1 |
| Ott et al. (ISAR-PEBIS) | PTA vs. DCB | Apr-10 | Dec-13 | 34 | 36 | 2017 | 0 | 1 | 1 | 0 | 0 | 1 | 1 | 0 | 0 |
| Wyttenbach et al. | PTA vs. Brachy | NA | NA | 10 | 10 | 2007 | 0 | 1 | 0 | 0 | 0 | 0 | 0 | 0 | 0 |
| Tepe et al. (THUNDER trial) | PTA vs. DCB | Jun-04 | Jun-05 | 54 | 48 | 2008 | 0 | 0 | 0 | 0 | 0 | 1 | 0 | 0 | 0 |
| Werk et al. | PTA vs. DCB | Jul-04 | Jan-06 | 42 | 45 | 2008 | 0 | 0 | 1 | 1 | 0 | 1 | 0 | 0 | 0 |
| Buszman et al. (BIOPAC trial) | PTA vs. DCB | Sep-14 | Sep-17 | 33 | 33 | 2018 | 1 | 0 | 1 | 1 | 0 | 1 | 1 | 0 | 0 |
| Jia et al (AcoArt trial) | PTA vs. DCB | Apr-13 | Jun-14 | 100 | 100 | 2016 | 1 | 0 | 1 | 1 | 1 | 1 | 1 | 0 | 0 |
| Kinstner et al. (PACUBA trial) | PTA vs. DCB | Nov-10 | Nov-13 | 39 | 35 | 2016 | 1 | 1 | 0 | 1 | 1 | 1 | 0 | 0 | 0 |
| Krishnan et al. (ILLUMENATE trial) | PTA vs. DCB | Jun-13 | Jul-15 | 100 | 200 | 2017 | 1 | 1 | 1 | 1 | 0 | 1 | 1 | 0 | 1 |
| Rosenfield et al. | PTA vs. DCB | Aug-11 | Jul-12 | 160 | 316 | 2015 | 1 | 1 | 1 | 1 | 0 | 1 | 1 | 1 | 1 |
| Sachar et al (RANGER II SFA trial) | PTA vs. DCB | Mar-17 | Aug-18 | 98 | 278 | 2021 | 1 | 1 | 1 | 1 | 1 | 1 | 1 | 0 | 0 |
| Scheinert et al. (BIOLUX trial) | PTA vs. DCB | Oct-10 | Aug-11 | 30 | 30 | 2015 | 0 | 1 | 1 | 0 | 1 | 1 | 1 | 0 | 0 |
| Scheinert et al. (LEVANT I trial) | PTA vs. DCB | Jun-09 | Dec-09 | 52 | 49 | 2014 | 1 | 1 | 1 | 1 | 0 | 1 | 1 | 1 | 0 |
| Schroeder et al. (ILLUMENATE European trial) | PTA vs. DCB | Dec-12 | Apr-15 | 72 | 222 | 2017 | 1 | 1 | 1 | 1 | 1 | 1 | 1 | 0 | 0 |
| Steiner et al. | PTA vs. DCB | Jan-14 | Oct-15 | 34 | 71 | 2018 | 1 | 1 | 1 | 1 | 0 | 1 | 1 | 1 | 0 |
| Teichgraber et al. | PTA vs. DCB | Sep-15 | Dec-16 | 86 | 85 | 2020 | 1 | 0 | 0 | 1 | 1 | 1 | 1 | 1 | 1 |
| Tepe et al. (CONSEQUENT trial) | PTA vs. DCB | Nov-13 | Apr-15 | 75 | 78 | 2017 | 1 | 1 | 0 | 1 | 0 | 1 | 1 | 0 | 0 |
| Tepe et al. (COPA COBANA trial) | PTA vs. DCB | Nov-11 | Dec-13 | 41 | 47 | 2020 | 0 | 0 | 1 | 0 | 1 | 1 | 1 | 0 | 0 |
| Tepe et al. (IN.PACT SFA trial) | PTA vs. DCB | Sep-10 | Apr-11 | 111 | 220 | 2015 | 1 | 1 | 1 | 1 | 0 | 1 | 1 | 0 | 1 |
| Werk et al. (PACIFER trial) | PTA vs. DCB | Mar-10 | Aug-11 | 47 | 44 | 2012 | 0 | 1 | 1 | 0 | 0 | 1 | 1 | 0 | 0 |
| Ye et al. | PTA vs. DCB | Jul-14 | Apr-17 | 100 | 100 | 2021 | 0 | 1 | 1 | 0 | 1 | 1 | 1 | 0 | 0 |
| Dake et al. (Zilver PTX trial) | PTA vs. DES | Mar-05 | Aug-08 | 238 | 236 | 2011 | 1 | 0 | 1 | 1 | 0 | 1 | 1 | 0 | 0 |
| Dippel et al. (EXCITE ISR trial) | PTA vs. PTA+A | Jun-11 | Feb-14 | 81 | 169 | 2015 | 1 | 1 | 0 | 1 | 0 | 1 | 0 | 0 | 0 |

NA = Not available

**Supplemental Table S2. Risk of bias.**

| **Study ID** | **D1** | **D2** | **D3** | **D4** | **D5** | **Overall** |  |  |
| --- | --- | --- | --- | --- | --- | --- | --- | --- |
| Goueffic et al. (EMINENT) |  |  |  |  |  |  |  | Low risk |
| Krankenberg et al. (FAST) |  |  |  |  |  |  |  | Some concerns |
| Krankenberg et al. (FAIR) |  |  |  |  |  |  |  | High risk |
| Krishnan et al. (ILLUMENATE) |  |  |  |  |  |  |  |  |
| Minar et al. |  |  |  |  |  |  | D1 | Randomization process |
| Ott et al. (ISAR-PEBIS) |  |  |  |  |  |  | D2 | Deviations from the intended interventions |
| Schroeder et al. (ILLEMENATE euro) |  |  |  |  |  |  | D3 | Missing outcome data |
| Tepe 2015 (IN.PACT SFA) |  |  |  |  |  |  | D4 | Measurement of the outcome |
| Therasse et al. |  |  |  |  |  |  | D5 | Selection of the reported result |
| Werk et at. |  |  |  |  |  |  |  |  |
| Amighi et al. |  |  |  |  |  |  |  |  |
| Rosenfield et al. |  |  |  |  |  |  |  |  |
| Schillinger et al. |  |  |  |  |  |  |  |  |
| Tepe et al. (THUNDER) |  |  |  |  |  |  |  |  |
| Bosiers et al. |  |  |  |  |  |  |  |  |
| de Boer et al. (RAPID) |  |  |  |  |  |  |  |  |
| Iida et al. (MDT-2113 SFA) |  |  |  |  |  |  |  |  |
| Iida et al. (SM-01) |  |  |  |  |  |  |  |  |
| Scheinert et al. |  |  |  |  |  |  |  |  |
| Tepe et al. (COPA COBANA) |  |  |  |  |  |  |  |  |
| Geraghty et al. (VIBRANT) |  |  |  |  |  |  |  |  |
| Bausback et al. |  |  |  |  |  |  |  |  |
| Lammer et al. (VIASTAR) |  |  |  |  |  |  |  |  |
| Liistro et al. (DEBATE-SFA) |  |  |  |  |  |  |  |  |
| Cai et al. |  |  |  |  |  |  |  |  |
| Fanelli et al. (DEBELLUM) |  |  |  |  |  |  |  |  |
| Liao et al. |  |  |  |  |  |  |  |  |
| Ye et al. |  |  |  |  |  |  |  |  |
| Buszman et al. (BIOPAC) |  |  |  |  |  |  |  |  |
| Dippel et al. (EXCITE ISR) |  |  |  |  |  |  |  |  |
| Goueffic et al. (BATTLE) |  |  |  |  |  |  |  |  |
| Jia et al. (AcoArt) |  |  |  |  |  |  |  |  |
| Kinstner et al. (PACUBA) |  |  |  |  |  |  |  |  |
| Liistro et al. (DRASTICO) |  |  |  |  |  |  |  |  |
| Sachar et al. (RANGER II SFA) |  |  |  |  |  |  |  |  |
| Scheinert et al. (LEVANT 1) |  |  |  |  |  |  |  |  |
| Steiner et al. |  |  |  |  |  |  |  |  |
| Dake (Zilver PTX) |  |  |  |  |  |  |  |  |
| Laird et al. (RESILIENT) |  |  |  |  |  |  |  |  |
| Therasse et al. (2016) |  |  |  |  |  |  |  |  |
| Werk et al. (PACIFER) |  |  |  |  |  |  |  |  |
| Zeller et al. (DEFINITIVE AR) |  |  |  |  |  |  |  |  |
| Teichgraber et al. |  |  |  |  |  |  |  |  |
| Wyttenbach et al. |  |  |  |  |  |  |  |  |
| Becquemin et al. |  |  |  |  |  |  |  |  |
| Shammas et al. (JET-RANGER) |  |  |  |  |  |  |  |  |
| Diehm et al. |  |  |  |  |  |  |  |  |
| Duda et al. (SIROCOO) |  |  |  |  |  |  |  |  |
| Krueger et al. |  |  |  |  |  |  |  |  |
| Pokrajac et al. (Vienna-3) |  |  |  |  |  |  |  |  |
| Wolfram et al. (Vienna-5) |  |  |  |  |  |  |  |  |
| Chalmers et al. (SUPER) |  |  |  |  |  |  |  |  |
| Tepe et al. (Disrupt PAD II) |  |  |  |  |  |  |  |  |
| Poncyljusz et al. |  |  |  |  |  |  |  |  |
| Tacke et al. (FREEWAY) |  |  |  |  |  |  |  |  |
| Tepe 2017 (CONSEQUENT) |  |  |  |  |  |  |  |  |
| Dick et al. |  |  |  |  |  |  |  |  |

**Supplemental Table S3. Demographics and Procedural Characteristics by Intervention**

| Demographic | PTA | DCB | DCB+BMS | BMS | DES | CSG | L+DCB | A+DCB | PTA+A | Brachy | BMS+Brachy | CBA |
| --- | --- | --- | --- | --- | --- | --- | --- | --- | --- | --- | --- | --- |
| Age, y | 68.52 | 68.73 | 74 | 67.88 | 69.63 | 68.52 | 72.20 | 69.20 | 68.50 | 67.06 | 66.55 | 65.20 |
| Male sex, % | 65.55 | 65.54 | 75.5 | 68.86 | 72.87 | 68.63 | 69.30 | 70.43 | 62.70 | 64.98 | 72.00 | 55.00 |
| Body mass index, kg/m² | 27.40 | 27.28 |  | 26.53 | 27.95 |  |  | 29.90 | 28.30 | 26.90 | 27.90 | 26.80 |
| Hypertension, % | 77.93 | 82.08 | 88.7 | 78.33 | 77.57 | 79.90 | 94.80 | 85.47 | 95.80 | 69.14 | 74.50 | 94.00 |
| Dyslipidemia, % | 67.68 | 66.57 | 62.3 | 71.57 | 66.18 | 61.27 | 86.80 | 75.50 | 96.40 | 67.30 | 71.00 | 75.50 |
| Diabetes mellitus, % | 41.55 | 45.66 | 70.6 | 37.96 | 43.80 | 37.67 | 42.10 | 37.57 | 47.00 | 34.32 | 37.50 | 44.50 |
| Current smoking, % | 40.06 | 38.36 | 54.9 | 43.48 | 39.94 | 55.00 |  | 33.83 |  | 35.47 | 43.00 | 44.00 |
| Heart failure, % | 6.28 | 5.63 |  | 7.70 | 6.90 | 4.20 |  | 2.10 |  |  |  |  |
| Coronary artery disease, % | 47.24 | 42.07 | 35.3 | 41.98 | 31.43 | 22.00 |  | 41.50 | 64.30 | 37.50 | 39.50 | 60.00 |
| Prior myocardial infarction, % | 18.46 | 17.54 |  | 19.10 | 16.00 | 23.60 | 25.80 | 18.80 |  |  |  | 20.00 |
| Carotid artery disease, % | 21.15 | 24.80 |  |  |  |  |  |  |  |  |  |  |
| Prior CVA (stroke/TIA), % | 12.93 | 15.48 |  | 10.40 | 11.67 | 5.60 | 12.60 | 15.95 | 10.70 | 15.00 | 5.00 | 20.00 |
| Renal insufficiency, % | 11.13 | 13.48 |  | 13.94 | 12.10 | 5.10 | 24.30 | 14.67 |  |  |  |  |
| End-stage renal disease, % |  | 0.00 | 9.4 | 6.45 |  | 17.00 |  | 0.00 |  |  |  |  |
| Target leg ankle-brachial index | 0.65 | 0.66 | 0.33 | 0.62 | 0.67 | 0.58 | 0.74 | 0.61 | 0.60 | 0.63 | 0.64 | 0.63 |
| Rutherford class, % |  |  |  |  |  |  |  |  |  |  |  |  |
| 1 | 5.52 | 1.00 |  | 2.67 | 2.03 |  |  |  |  | 40.00 |  |  |
| 2 | 24.95 | 23.12 |  | 32.06 | 34.78 | 23.90 | 16.30 | 16.80 |  | 3.75 |  | 13.00 |
| 3 | 64.97 | 63.86 | 20.8 | 60.73 | 51.52 | 60.70 | 77.80 | 62.97 |  | 81.23 |  | 73.00 |
| 4 | 9.47 | 9.69 | 20.8 | 7.21 | 6.96 | 9.15 | 5.90 | 10.60 |  | 7.00 |  | 6.50 |
| 5 | 5.21 | 7.66 | 54.7 | 9.12 | 15.63 | 6.80 | 0.00 | 6.60 |  | 12.00 |  |  |
| 6 | 0.08 | 0.62 | 3.7 | 4.85 | 0.00 |  |  |  |  |  |  |  |
| Chronic limb-threatening ischemia (Rutherford 4-6), % | 15.74 | 15.93 | 79.2 | 25.10 | 28.10 | 12.80 | 5.90 | 12.90 | 16.00 | 34.27 | 13.00 | 13.50 |
| Prior interventions, % | 59.75 | 18.50 |  | 56.05 |  |  |  | 29.20 |  |  |  |  |
| Percutaneous transluminal angioplasty | 13.13 | 20.50 |  |  |  |  |  |  |  |  |  |  |
| Drug-coated balloon | 2.00 | 1.10 |  |  |  |  |  |  |  |  |  |  |
| Stent | 37.67 | 41.03 |  |  |  |  |  |  |  |  |  |  |
| Atherectomy | 3.10 | 2.50 |  |  |  |  |  |  |  |  |  |  |
| Lesion characteristics |  |  |  |  |  |  |  |  |  |  |  |  |
| No. of lesions treated | 1.15 | 1.21 |  | 1.14 |  |  |  |  |  |  |  |  |
| De novo lesions, % | 74.47 | 74.97 | 100 | 96.48 | 98.17 | 66.67 | 100.00 | 95.07 | 0.00 | 82.77 | 91.50 | 100.00 |
| In-stent restenosis, % | 24.16 | 27.37 | 0 | 0.00 | 0.00 | 33.33 | 0.00 | 0.00 | 100.00 | 1.20 | 0.00 | 0.00 |
| No. of patient runoff vessels, % |  |  |  |  |  |  |  |  |  |  |  |  |
| 0 | 1.78 | 4.47 |  | 13.58 | 11.55 | 44.00 |  | 16.10 |  |  | 10.00 |  |
| 1 | 20.06 | 22.74 |  | 19.90 | 16.50 | 27.15 |  | 30.80 |  |  | 9.00 | 17.00 |
| 2 | 43.44 | 44.74 |  | 22.82 | 32.85 | 32.00 |  | 27.35 |  |  | 17.00 | 33.00 |
| 3 | 43.28 | 32.92 |  | 50.99 | 42.65 | 18.85 |  | 24.15 |  |  | 63.00 | 50.00 |
| Popliteal involvement, % | 16.66 | 15.91 | 25.5 | 11.44 | 12.86 |  | 18.30 | 26.83 | 21.30 | 28.37 |  |  |
| Lesion length, cm | 8.45 | 9.78 | 9.4 | 9.98 | 9.67 | 18.40 | 10.09 | 11.12 | 19.60 | 5.51 | 9.70 | 2.57 |
| Total occlusion, % | 28.38 | 32.39 | 54.5 | 50.79 | 49.15 | 54.40 | 26.30 | 23.80 | 30.50 | 21.40 | 46.50 | 19.50 |
| Diameter stenosis at baseline, % |  |  |  |  |  |  |  |  |  |  |  |  |
| mean | 80.25 | 82.72 | 91 | 78.77 | 84.37 |  | 85.00 | 79.50 | 81.70 | 83.10 | 88.00 | 90.00 |
| Calcification, % | 44.70 |  |  | 56.80 |  |  |  |  |  |  |  |  |
| None | 38.58 | 32.80 | 60 | 33.66 | 7.35 | 43.05 |  | 17.85 |  |  | 31.00 | 17.00 |
| Mild/moderate | 47.15 | 41.30 | 18.2 | 47.97 | 57.10 | 61.20 |  |  |  |  |  |  |
| Severe | 14.89 | 21.55 | 21.8 | 20.64 | 34.93 | 17.20 | 82.90 | 25.00 | 3.90 |  | 23.00 |  |
| Procedural characteristics |  |  |  |  |  |  |  |  |  |  |  |  |
| Procedure time, min | 66.75 | 63.13 |  | 53.00 | 52.00 | 62.00 | 89.90 |  |  |  |  |  |
| No. of treatment balloons | 1.07 | 1.41 |  |  |  |  | 1.60 |  |  |  |  |  |
| Procedural complication, % |  |  |  |  |  |  |  |  |  |  |  |  |
| Any dissection | 41.95 | 48.11 |  | 2.40 | 18.35 |  | 16.10 | 54.80 | 7.70 |  |  | 13.30 |
| Flow-limiting dissection (≥type D) | 5.53 | 6.69 |  |  | 0.00 | 0.00 | 1.40 | 4.33 |  |  |  | 0.00 |
| Thrombus | 2.43 | 2.97 |  | 1.83 | 6.10 |  |  |  |  |  | 17.00 |  |
| Aneurysm/pseudoaneurysm | 1.43 | 0.10 |  | 2.03 |  | 1.30 |  | 6.30 |  | 0.90 | 7.10 | 4.80 |
| Perforation | 0.45 | 0.84 |  |  | 0.00 |  | 0.00 | 2.10 |  |  |  |  |
| Distal embolus | 1.10 | 1.21 |  | 0.58 | 1.30 | 2.75 | 0.00 | 2.10 | 8.30 | 6.70 | 2.40 | 4.80 |
| Diameter stenosis after intervention, % | 22.85 | 23.30 |  | 13.45 | 15.60 |  | 21.50 | 19.60 | 23.90 | 24.80 |  |  |

**Supplemental Table S4. Prediction Intervals of Primary Endpoints using PTA as the Comparator**

| **Technical Success** | |
| --- | --- |
| **Endovascular Intervention vs. PTA** | **95% Prediction Interval** |
| A+DCB | (0.75, 23.66) |
| BMS | (1.46, 36.38) |
| Brachy | (0.11, 9.04) |
| CBA/A | (0.34, 462.69) |
| CSG | (1.60, 135.57) |
| DCB | (0.37, 5.52) |
| DCB+BMS | (0.03, 66.99) |
| DES | (0.59,32.05) |
| L+DCB | (0.41, 17.75) |
| **12-month Primary Patency** | |
| **Endovascular Intervention vs. PTA** | **95% Prediction Interval** |
| A+DCB | (1.17, 18.28) |
| BMS | (1.34, 10.85) |
| BMS+Brachy | (1.42, 37.42) |
| Brachy | (0.56, 10.2) |
| CSG | (1.61, 17.82) |
| DCB | (1.39, 9.09) |
| DCB+BMS | (1.73, 24.10) |
| DES | (2.02, 17.49) |
| L+DCB | (1.66, 28.84) |
| PTA+A | (0.82, 15.83) |

**Supplemental Table S5. Meta-Regression of groups of interest in Percutaneous Transluminal Angioplasty vs. Drug-coated Balloon Studies**

|  | Technical Success | | | Primary Patency at 12 Months | | | Improvement in Rutherford at 12 Months | | | TLR at 12 Month | | | All Cause Death at 12 Months | | |
| --- | --- | --- | --- | --- | --- | --- | --- | --- | --- | --- | --- | --- | --- | --- | --- |
| Subgroup | **Coefficient** | **CI** | **P-value** | **Coefficient** | **CI** | **P-value** | **Coefficient** | **CI** | **P-value** | **Coefficient** | **CI** | **P-value** | **Coefficient** | **CI** | **P-value** |
| Age | 0.9865 | (0.0697, 1.3962) | 0.9390 | 1.0369 | (0.8819, 1.2193) | 0.6609 | 1.1756 | (0.8245, 1.6764) | 0.3714 | 0.9076 | (0.76614, 1.0752) | 0.2621 | 0.9772 | (0.8556, 1.1160) | 0.7332 |
| Female | 0.0006 | (0.0000, 2.0792) | 0.0746 | 0.4835 | (0.0105, 22.3647) | 0.7103 | 0.0012 | (0.0000, 0.7360) | **0.0400** | 1.4962 | (0.0293, 76.3343) | 0.8408 | 22.0067 | (0.8478, 571.2474) | 0.0628 |
| Diabetes | 1.0151 | (0.9422, 1.0934) | 0.0694 | 0.9972 | (0.9563, 1.0340) | 0.8967 | 1.0110 | (0.9462, 1.0804) | 0.7455 | 0.9807 | (0.9443, 1.0186) | 0.3137 | 0.9832 | (0.9535, 1.0140) | 0.2817 |
| Smoker | 1.0394 | (0.9757, 1.1073) | 0.2313 | 0.9718 | (0.9277, 1.0181) | 0.2286 | 1.0126 | (0.9420, 1.0885) | 0.7347 | 1.0263 | (0.9894, 1.0645) | 0.1645 | 0.9919 | (0.9582, 1.0266) | 0.6421 |
| Renal Insufficiency | 1.0566 | (0.9431, 1.1839) | 0.3424 | 0.9867 | (0.9020, 1.0792) | 0.7693 | 0.9985 | (0.9149, 1.0897) | 0.9727 | 1.0305 | (0.9240, 1.1493) | 0.5894 | 0.9890 | (0.9257, 1.0567) | 0.7441 |
| CLTI | 0.7470 | (0.1331, 4.1925) | 0.7403 | 1.6618 | (0.7207, 3.8321) | 0.2335 | 1.8858 | (0.2971, 11.9712) | 0.5011 | 0.4071 | (0.1583, 1.0469) | 0.0622 | 1.1295 | (0.4605, 2.7705) | 0.7903 |
| Total Occlusion | 0.9634 | (0.9163, 1.0128) | 0.1441 | 0.9925 | (0.9639, 1.0219) | 0.6138 | 1.0050 | (0.9549, 1.0584) | 0.2006 | 1.0186 | (0.9902, 1.0479) | 0.2009 | 1.0232 | (1.0061, 1.0406) | **0.0076** |
| Calcification: Severe | 1.0162 | (0.9755, 1.0586) | 0.4412 | 0.9987 | (0.9698, 1.0286) | 0.9336 | 0.8714 | (0.7708, 0.9851) | **0.0278** | 0.9984 | (0.9667, 1.0312) | 0.9245 | 1.0072 | (0.9850, 1.0299) | 0.5287 |
| De-Novo Lesions | 0.9989 | (0.9852, 1.0127) | 0.8722 | 1.0073 | (0.9992, 1.0155) | 0.0780 | 1.0187 | (1.0104, 1.0271) | **<0.0001** | 0.9910 | (0.9838, 0.9981) | **0.0137** | 0.9951 | (0.9901, 1.002) | 0.0598 |

Meta-regression coefficients for various groups of interest in Percutaneous Transluminal Angioplasty (PTA) vs. Drug-Coated Balloon (DCB) studies.

CLI = chronic limb-threatening ischemia

**Supplemental Figure S1**

**
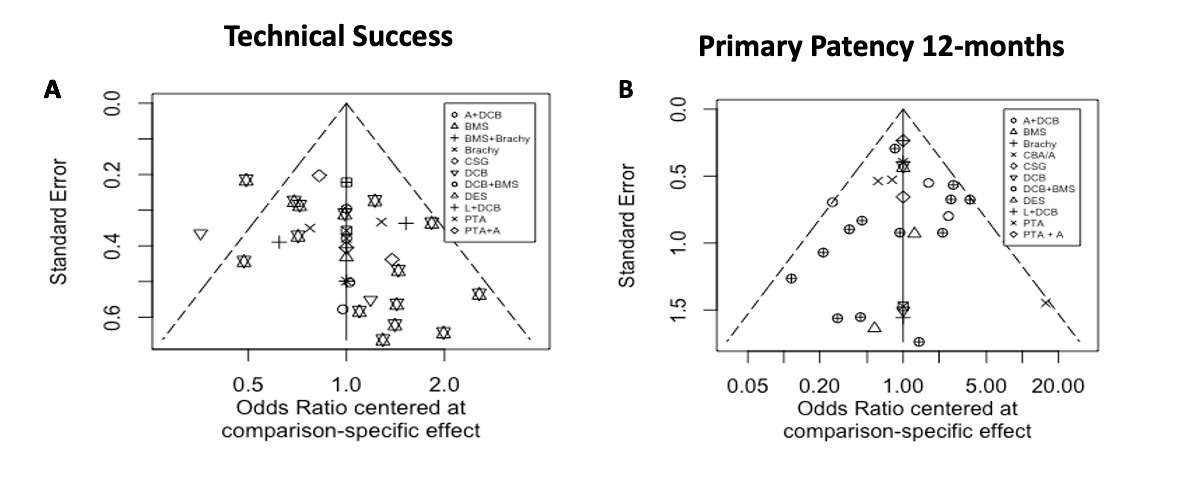
**

**Supplemental Figure S2.**

**
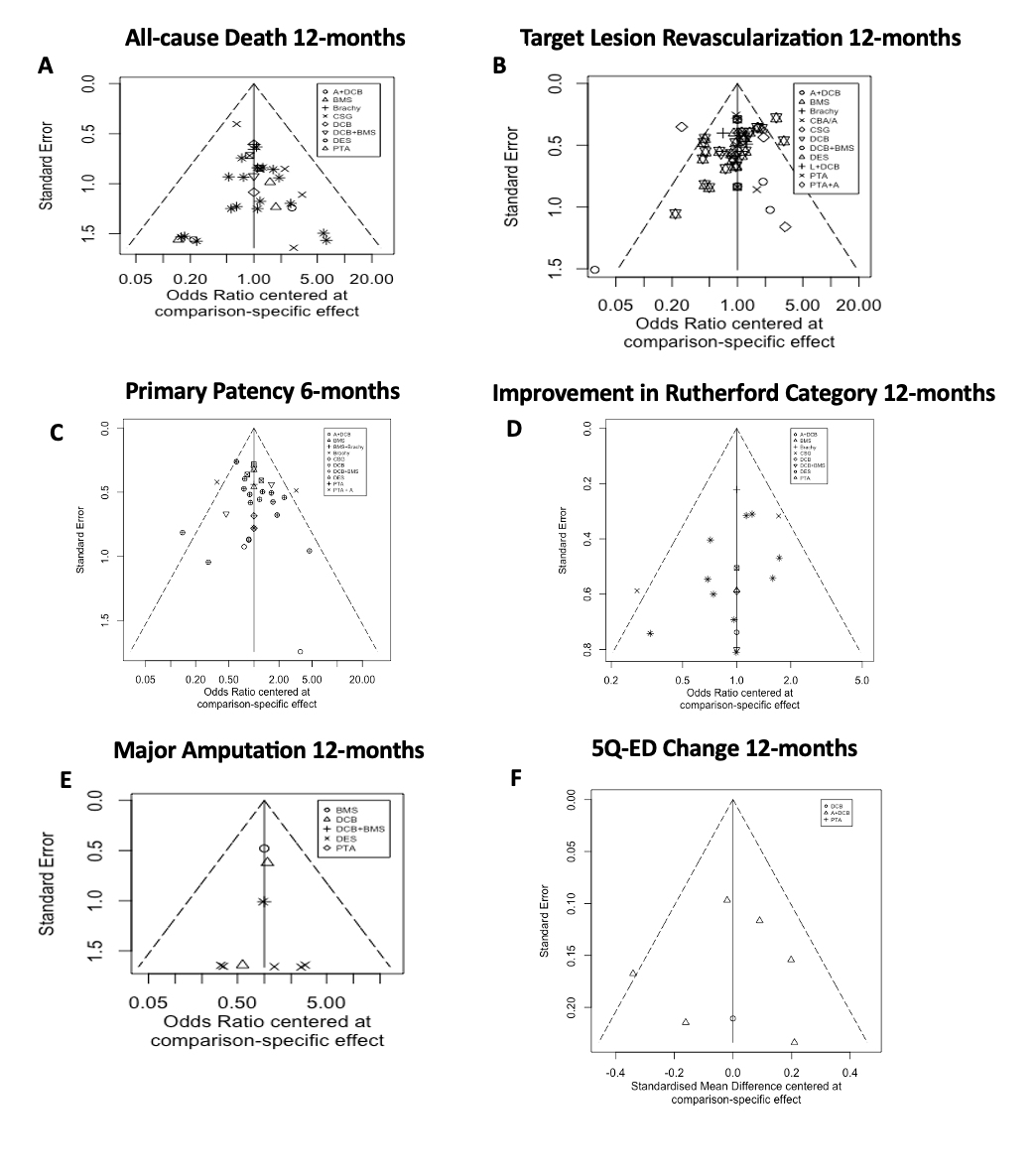
**

**Supplemental Figure S3**

**
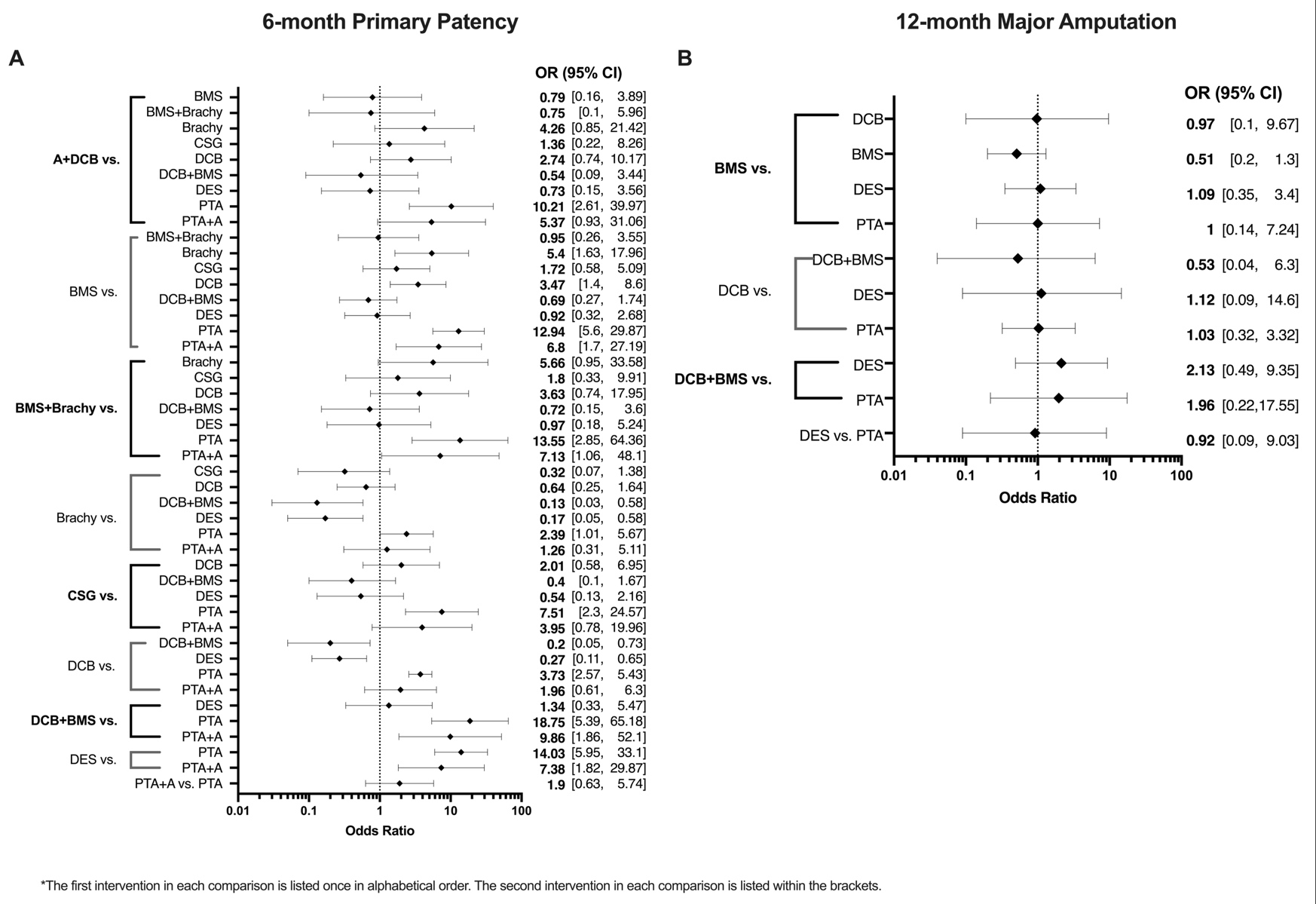
**

**Supplemental Figure S4**

**
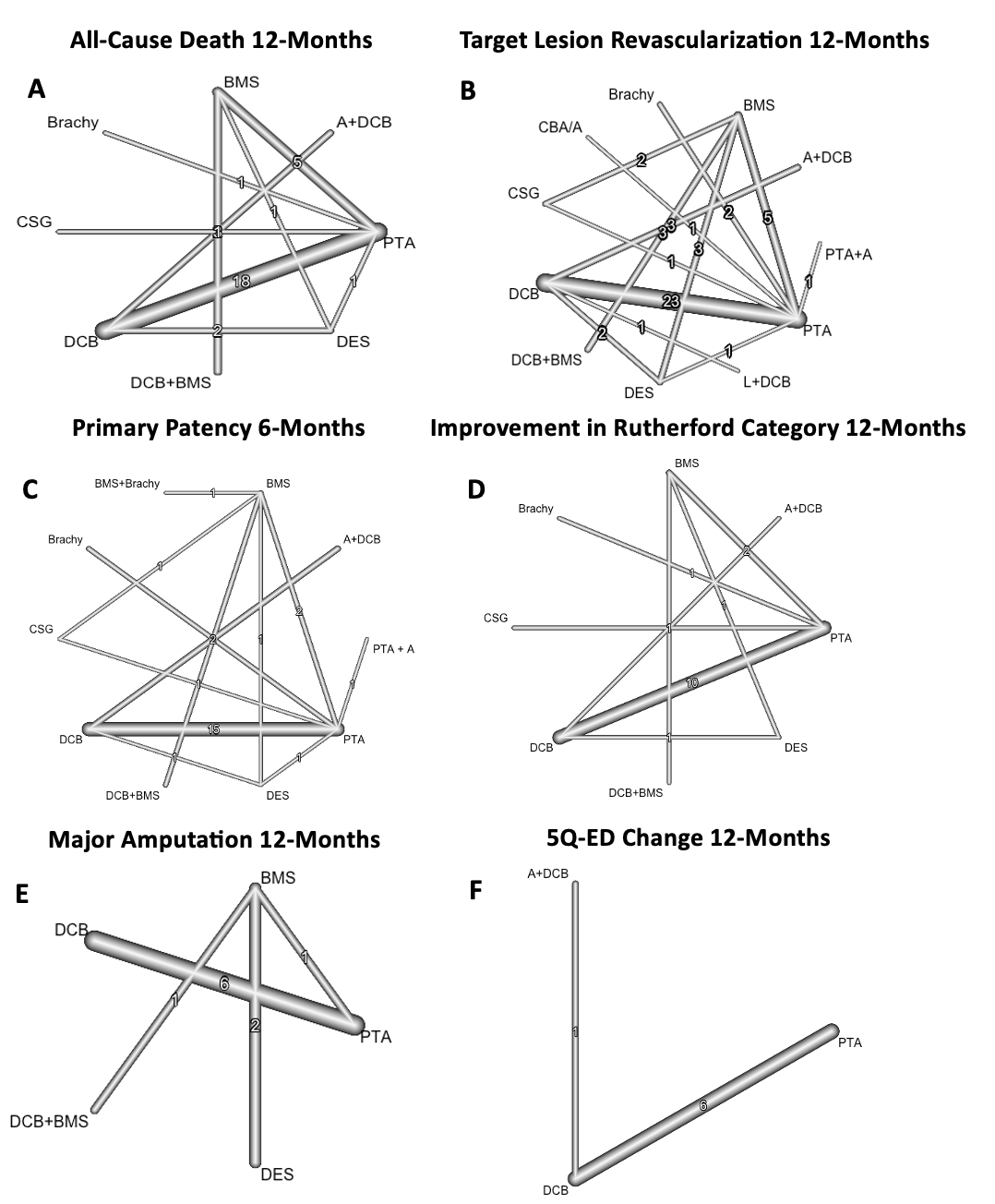
**
